# High FIB4 index is an independent risk factor of diabetic kidney disease in type 2 diabetes

**DOI:** 10.1038/s41598-021-88285-6

**Published:** 2021-06-03

**Authors:** Haruka Saito, Hayato Tanabe, Akihiro Kudo, Noritaka Machii, Moritake Higa, Satoshi Yamaguchi, Gulinu Maimaituxun, Kazumichi Abe, Atsushi Takahashi, Kenichi Tanaka, Koichi Asahi, Hiroaki Masuzaki, Hiromasa Ohira, Junichiro J. Kazama, Michio Shimabukuro

**Affiliations:** 1 Department of Diabetes, Endocrinology and Metabolism, Fukushima Medical University, 1 Hikarigaoka, Fukushima, Fukushima 960-1295 Japan; 2 Diabetes and Lifestyle-Related Disease Center, Tomishiro Central Hospital, Okinawa, Japan; 3 Department of Gastroenterology, Fukushima Medical University, Fukushima, Japan; 4 Department of Nephrology and Hypertension, Fukushima Medical University, Fukushima, Japan; 5 Department of Cardiology, Nakagami Hospital, Okinawa, Japan; 6 Division of Nephrology and Hypertension, Iwate Medical University, Iwate, Japan; 7 Division of Endocrinology, Diabetes and Metabolism, Hematology, Rheumatology (Second Department of Internal Medicine), University of the Ryukyu, Okinawa, Japan

**Keywords:** Diabetes complications, Non-alcoholic fatty liver disease, Chronic kidney disease

## Abstract

Nonalcoholic fatty liver disease (NAFLD) and nonalcoholic steatohepatitis (NASH) may be linked to development of chronic kidney diseases (CKD). The FIB4 index, a noninvasive liver fibrosis score, has been reported to predict CKD in non-diabetic patients, but there are no reports yet in diabetic cases. Therefore, we evaluated the prognostic impact of FIB4 index on the risk of developing diabetic kidney disease (DKD) in Japanese patients with type 2 diabetes in a retrospective cohort study. We assessed patients with type 2 diabetes with an eGFR ≥ 60 mL/min/1.73 m^2^ and without dipstick positive proteinuria (≥ 1 +) at their first visit to our department. Participants were divided into two groups based on the FIB4 index at their first visit: FIB4 index > 1.3 and FIB4 index ≤ 1.3. The primary endpoint was defined as a decrease in eGFR < 60 mL/min/1.73 m^2^ or the onset of proteinuria during the course of treatment. The average age of all 584 type 2 diabetic participants (360 [61.6%] men) was 55 ± 11 years. There were 187 patients in the FIB4 index group > 1.3 (32.0%) and the median observation period was 6.0 (3.8–11.0) years. Kaplan–Meier survival analysis indicated that the risks of developing DKD, eGFR < 60 and proteinuria were all higher in FIB4 index > 1.3 patients than in FIB4 ≤ 1.3 patients. In the Cox regression analysis, an FIB4 index > 1.3 was a significant predictor for onset of DKD (HR 1.54, 95% CI 1.15–2.08) and proteinuria (HR 1.55, 95% CI 1.08–2.23), but not for an eGFR < 60 (HR 1.14, 95% CI 0.79–1.99). To the best of our knowledge, this is the first study to demonstrate that an FIB4 index > 1.3 has a prognostic impact on the development of CKD and proteinuria in type 2 diabetic patients. This warrants further investigation of the prognostic impact of the development of DKD or proteinuria.

## Introduction

Chronic kidney disease (CKD) associated with diabetes mellitus, often referred as diabetic kidney disease (DKD)^1–4^, is the leading cause of end-stage kidney disease (ESKD) for patients with diabetes^5^.The treatment of earlier stages of DKD is effective in slowing the progression toward ESRD^1–3^. Thus, early detection of precursors and/or risk factors for DKD is crucial^1–3^. Family history of DKD, smoking history, and control of glycemic, blood pressure, and plasma lipid levels are established factors for identifying people at a greater risk of DKD development and progression. Among emerging risk markers for CKD^1–4^, nonalcoholic fatty liver disease (NAFLD) is also an exacerbation factor for the development and progression of CKD in the non-diabetic^6–8^ and diabetic populations^9–13^.

NAFLD moves pathologically from non-alcoholic fatty liver (NAFL) to non-alcoholic steatohepatitis (NASH) and cirrhosis (LC)^14^. A liver biopsy is useful to detect progressive NASH in NAFLD patients for estimation of their prognosis. However, liver biopsy has limitations such as invasiveness, sampling errors, and cost. For this reason, multiple scoring systems that noninvasively predict the progression to NASH and liver fibrosis have been proposed^15–18^. The FIB4 index is a high ability non-invasive scoring system used to predict NASH and liver fibrosis^19–21^. A relationship between the FIB4 index and onset of CKD was reported in non-diabetic patients^22^, but the relationship has never been studied in a diabetic population.

Therefore, we evaluated the prognostic impact of the FIB4 index on the risk of developing DKD in Japanese type 2 diabetic patients in a single-center retrospective cohort study.

## Results

### General characteristics

**Figure 1:**
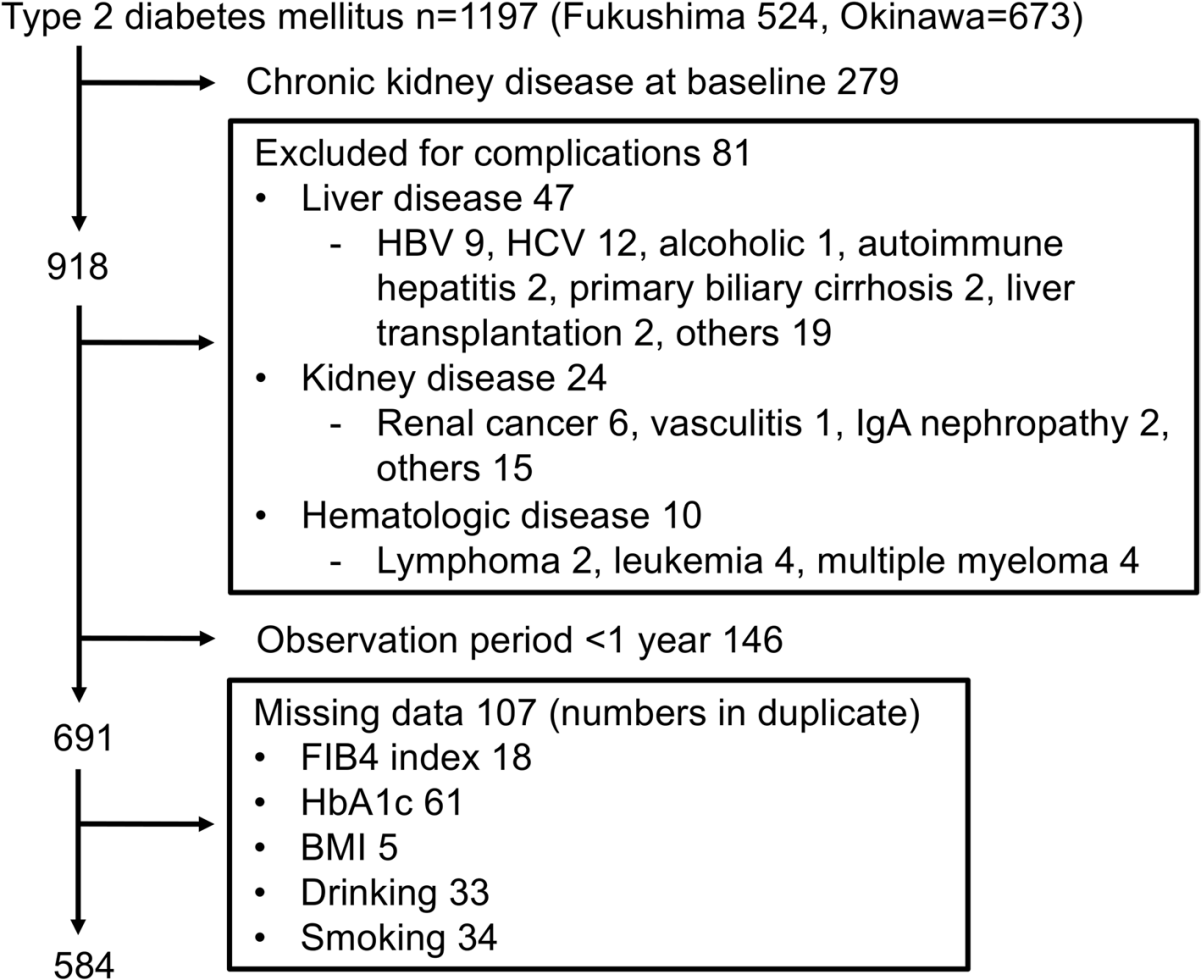
Flow chart for study recruitment. Total of 1197 patients with type 2 diabetes mellitus were selected from two Japanese centers on medical records. *HBV* hepatitis B virus hepatitis, *HCV* hepatitis C virus hepatitis. Patients diagnosed with liver cirrhosis and heavy drinker (consumption of ethanol less than 20 g/day for women and 30 g/day for men) had been excluded in advance.

Flow chart for study recruitment was shown in Fig. 1. Baseline characteristics of the patients are shown in Table 1. Among the 584 participants with type 2 diabetes mellitus, 187 (32.0%) were FIB4 index > 1.3 and 397 (68.0%) were FIB4 index ≤ 1.3. FIB4 index > 1.3 had a higher age (62.0 ± 8.0 vs 52.2 ± 10.0, *P* < 0.001), a larger proportion of men (130 (69.1%) vs 230 (58.1%), *P* = 0.014), and a lower BMI (25.1 ± 3.8 vs 26.4 ± 5.7, *P* = 0.005). Past drinkers were higher in FIB4 index > 1.3, but past smokers were similar. The prevalence of dyslipidemia was lower in FIB4  index > 1.3, but that of hypertension was similar. Blood biochemistry showed that FIB4  index > 1.3 had significantly fewer white blood cells and platelets and lower eGFR. There was no significant difference in HbA1c, ALT and albumin. By contrast, AST and γGT were high and TC, TG and LDL-cholesterol were low in FIB4 index > 1.3. The decrease of eGFR < 60 mL/min/1.73 m^2^ [41.8% vs 26.8%, *P* = 0.001], and onset of DKD [58.5% vs 43.7%, *P* = 0.001] were higher in FIB4 index > 1.3 and that of proteinuria showed a non-significant difference (proteinuria 1 + (35.1% vs 28.3%, *P* = 0.09).

**Table 1:** Baseline characteristics of studied patients.

Parameters	All (n = 584)	FIB4 ≤ 1.3 (n = 397)	FIB4 > 1.3 (n = 187)	*P* value
Age, years	55.4 ± 10.5	52.2 ± 10.0	62.0 ± 8.0	< 0.01
Men, n (%)	360 (61.6)	230 (58.1)	130 (69.1)	< 0.05
Height, cm	160.6 ± 8.6	160.8 ± 8.8	160.0 ± 8.0	0.27
Body weight, kg	66.9 ± 13.5	67.9 ± 13.8	64.7 ± 12.4	< 0.01
Body mass index, kg/m^2^	26.0 ± 5.2	26.4 ± 5.7	25.1 ± 3.8	< 0.01
Past smoker, n (%)	286 (49.0)	197 (49.7)	89 (47.3)	0.59
Past drinker, n (%)	307 (52.6)	192 (48.5)	115 (61.2)	< 0.01
Dyslipidemia, n (%)	426 (72.9)	302 (76.3)	124 (66.0)	< 0.01
Hypertension, n (%)	359 (61.5)	233 (58.8)	126 (67.0)	0.06
**Drugs**				
Sulfonylurea, n (%)	92 (15.8)	56 (14.1)	36 (19.3)	0.11
Biguanide, n (%)	77 (13.2)	57 (14.4)	20 (10.7)	0.22
DPP4 inhibitor, n (%)	31 (5.3)	15 (3.8)	16 (8.6)	< 0.05
Thiazolidine, n (%)	27 (4.6)	18 (4.5)	9 (4.8)	0.88
αGI, n (%)	59 (10.1)	38 (9.6)	21 (11.2)	0.54
Glinide, n (%)	12 (2.1)	8 (2.0)	4 (0.7)	0.92
SGLT2 inhibitor, n (%)	2 (0.3)	1 (0.3)	1 (0.5)	0.59
Insulin, n (%)	36 (6.2)	22 (5.5)	14 (7.5)	0.36
ACEi/ARB, n (%)	113 (19.4)	74 (18.7)	39 (20.9)	0.54
White blood cell, /μL	5900 ± 2600	6200 ± 2800	5200 ± 2200	< 0.01
Hemoglobin, g/dL	14.1 ± 1.5	14.0 ± 1.5	14.2 ± 1.5	0.10
Platelet, 10^4^/μL	23.2 ± 6.7	25.7 ± 6.4	18.1 ± 4.0	< 0.01
Blood urea nitrogen, mg/dL	13.9 ± 3.9	13.7 ± 4.1	14.4 ± 3.5	< 0.05
Creatinine, mg/dL	0.70 ± 0.16	0.69 ± 0.16	0.72 ± 0.15	0.07
eGFR, mL/min/1.73 m^2^	81.9 ± 21.3	85.9 ± 18.8	79.6 ± 15.8	< 0.01
Hemoglobin A1c, %	7.7 ± 2.1	7.7 ± 2.1	7.6 ± 1.9	0.34
Aspartate aminotransferase (AST), U/L	21 (17–29)	20 (16–26)	25 (20–39)	< 0.01
Alanine aminotransferase (ALT), U/L	27 (19–41)	26 (19–39)	28 (20–45)	0.30
γ-Glutamyl transferase (γGT), U/L	34 (21–60)	32 (21–57)	40 (22–72)	< 0.05
Serum albumin, g/dL	4.3 ± 0.5	4.4 ± 0.5	4.3 ± 0.4	0.41
Total-cholesterol, mg/dL	203 ± 40	206 ± 40	197 ± 39	< 0.01
Triglyceride, mg/dL	130 (92–180)	139 (96–188)	117 (88–163)	< 0.01
HDL-cholesterol, mg/dL	51 ± 13	50 ± 13	53 ± 14	< 0.01
LDL-cholesterol, mg/dL	121 ± 36	123 ± 36	116 ± 34	< 0.05
FIB4 index	1.06 (0.77–1.44)	0.87 (0.67–1.07)	1.74 (1.46–2.20)	< 0.01
**Onset of DKD**				
Decrease of eGFR < 60, n (%)	182 (31.2)	106 (26.8)	76 (41.8)	< 0.01
Proteinuria (1 +) or more, n (%)	178 (30.5)	112 (28.3)	66 (35.1)	0.09
DKD, n (%)	283 (48.5)	173 (43.7)	110 (58.5)	< 0.01
Observation period, days	2,191 (1396–4015)	2,555 (1460–4322)	2,002 (1156–3517)	< 0.05

Data is presented as mean ± SD, median (25–75th percentile), or percentages.

*DKD* diabetic kidney disease, *DPP4* Dipeptidyl Peptidase-4, *αGI* -α-Glucosidase Inhibitor, *SGLT2* sodium glucose cotransporter 2, *ACEi/ARB* Angiotensin-converting enzyme inhibitor and angiotensin II receptor blocker, *HDL* high-density lipoprotein, *LDL* low-density lipoprotein, *UACR* urine albumin-to-creatinine ratio, *eGFR* estimated glomerular filtration rate.

### Univariate and multivariate analysis

**Figure 2:**
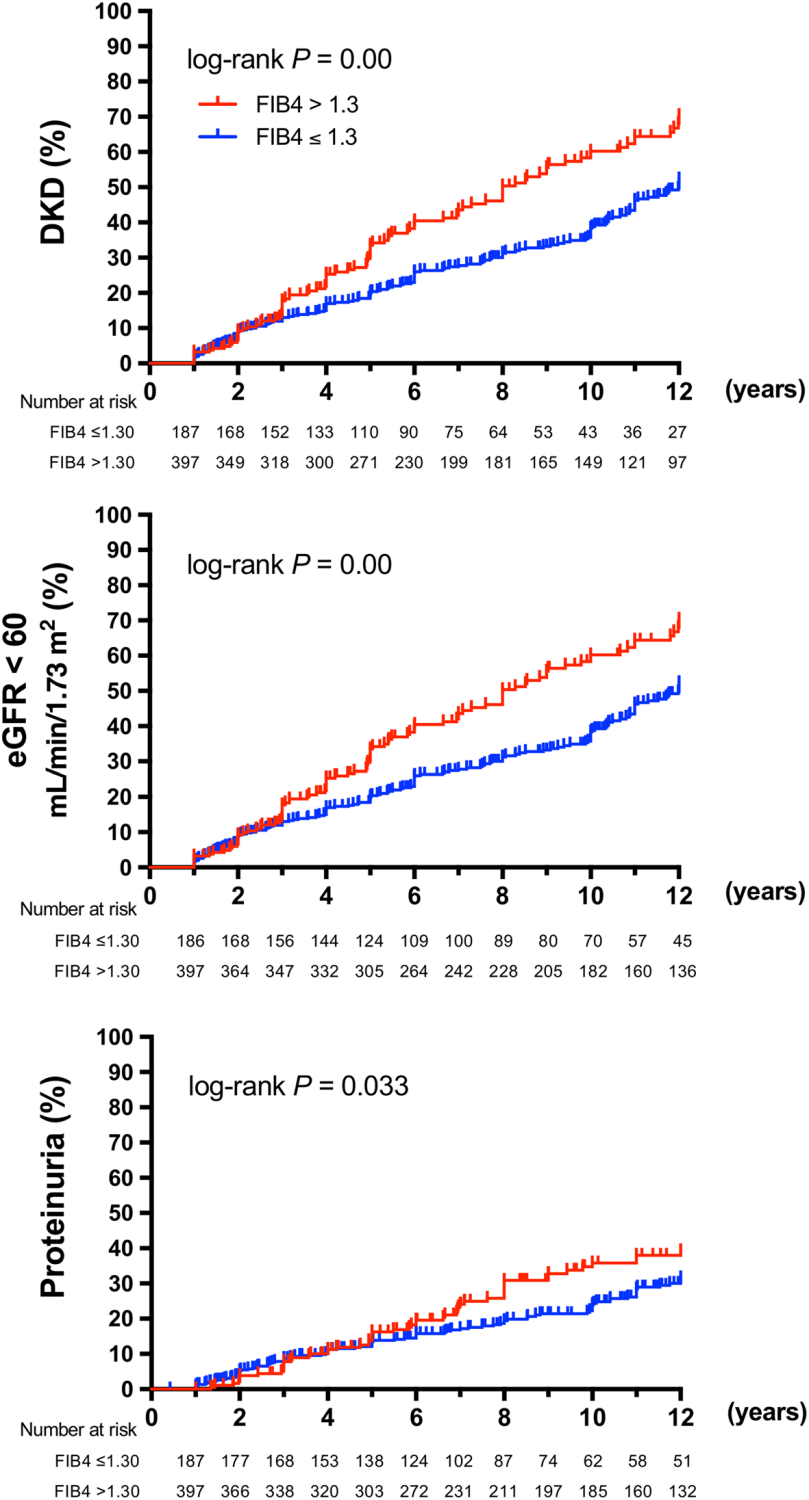
Kaplan Meier curves for the development of (**A**) diabetic kidney disease (DKD: eGFR < 60 mL/min/1.73 m^2^ or proteinuria), (**B**) eGFR < 60 mL/min/1.73 m^2^, and (**C**) proteinuria in type 2 diabetic patients with FIB4 index > 1.3 (red lines) or ≤ 1.3 (blue lines).

**Figure 3:**
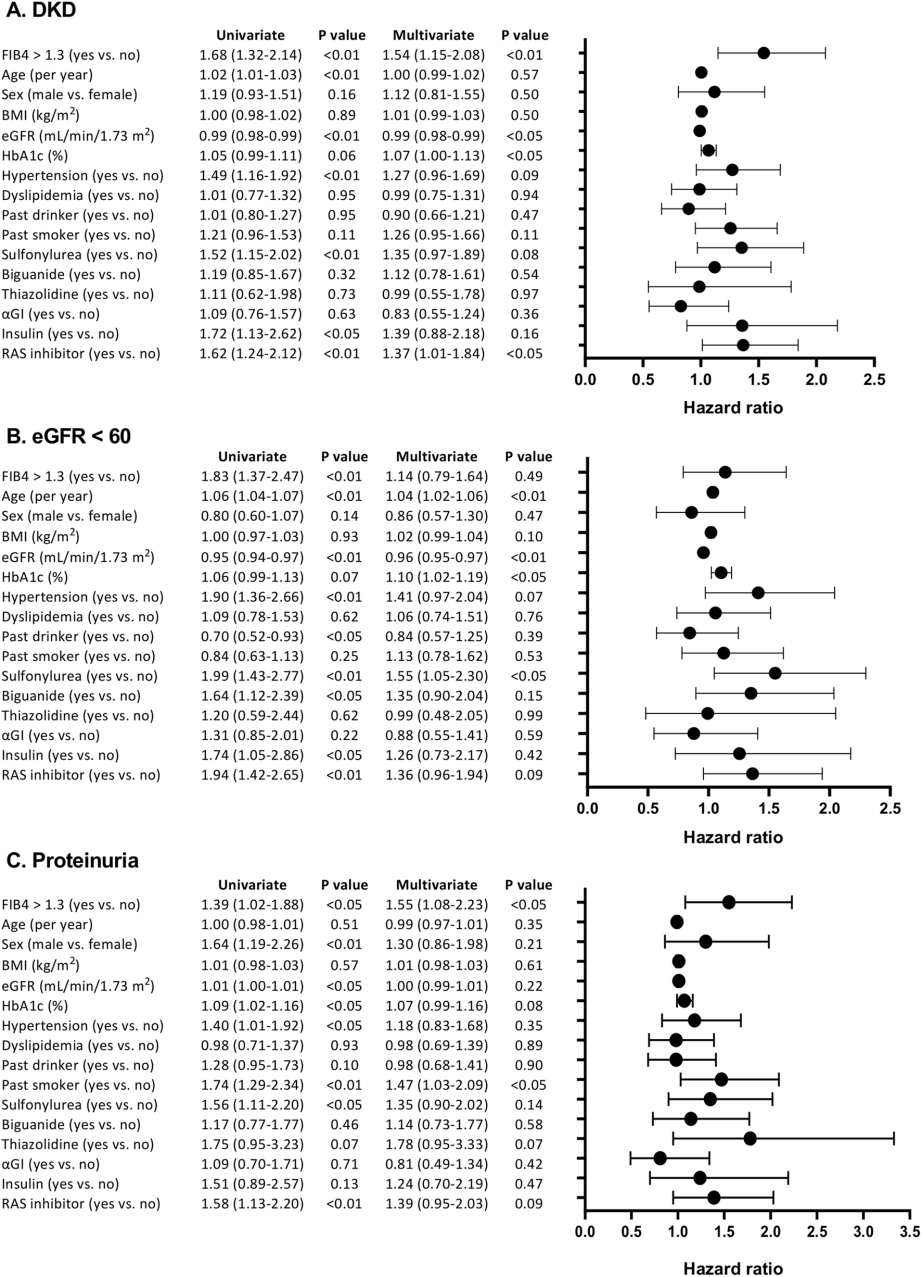
Univariate and Cox proportional hazard ratios of FIB4 index > 1.3 for the development of (**A**) diabetic kidney disease (DKD: eGFR < 60 mL/min/1.73 m^2^ or proteinuria), (**B**) eGFR < 60 mL/min/1.73 m^2^, and (**C**) proteinuria in type 2 diabetic patients. Cox proportional hazard models were adjusted for age, sex, BMI, baseline HbA1c, baseline eGFR, smoking and drinking status (current or past), comorbidities (hypertension, dyslipidemia) and anti-diabetic and anti-hypertensive medications. *95% CI* 95% confidence interval.

The results of Kaplan–Meier survival analysis are shown in Fig. 2. The median observation period was 6.0 (3.8–11.0) years. A total of 284 patients (incidence 48.6% [95% CI 44.6–52.7%], median 6.08 [3.88, 11.2] years) developed DKD, 182 (31.2% [27.8–35.3%], 8.72 [0.43, 12,6] years) patients developed DKD with eGFR < 60 mL/min/1.73 m^2^ and 178 (30.6% [95% CI 27.0–34.4%], 8.12 [5.07, 13.2] years) with proteinuria. The risk of developing DKD was higher in FIB4 ndex > 1.3 patients than in FIB4 INDEX ≤ 1.3 patients (Fig. 2A). Development of eGFR < 60 mL/min/1.73 m^2^ (Fig. 2B) and proteinuria (Fig. 2C) were also higher in FIB4 index > 1.3 patients. As shown in Fig. 3, FIB4  index > 1.3 was a significant variable for the onset of DKD (hazard ratio [HR] 1.68, 95% CI 1.32–2.14, *P* < 0.01) (Fig. 3A). Among other variables, baseline eGFR, baseline HbA1c and use of ACEi/ARB were significant risk variables for the onset of DKD. In the Cox proportional hazards model, adjusted for age, sex, BMI, baseline HbA1c, baseline eGFR, smoking and drinking status, comorbidities (hypertension, dyslipidemia), and anti-diabetic medications, FIB4 index > 1.3 was a significant variable for the onset of DKD (adjusted HR 1.54, 95% CI 1.15–2.08, *P* < 0.01). Because we could obtain HbA1c during follow-up only from one center (Fukushima Medical University), we calculated multivariate-adjusted analysis including median HbA1c during followup in a part of total participants (n = 307, Supplementary Fig. 1): FIB4 index > 1.3 and baseline eGFR were only a risk variable for the onset of DKD.

Development of proteinuria (Fig. 3C) was also higher in the FIB4 index > 1.3 patients (adjusted HR 1.55, 95% CI 1.08–2.23, *P* < 0.05). Past smoker was another independent variable for proteinuria. Meanwhile, FIB4 index > 1.3 was not a significant variable in the development of eGFR < 60 mL/min/1.73 m^2^ (Fig. 3B). Instead, age, baseline eGFR, baseline HbA1c and use of sulfonylurea were risk variables for the development of eGFR < 60.

We evaluated the impact of ultrasonography-determined NAFLD for the DKD hazard ratio in type 2 diabetic patients in whom abdominal ultrasonography could be performed (n = 96). Multivariate analysis showed that the presence of NAFLD was not a significant predictor for onset of DKD (odds ratio 0.71; 95% CI 0.37–1.36, P = 0.30).

### Sensitivity analysis

**Figure 4:**
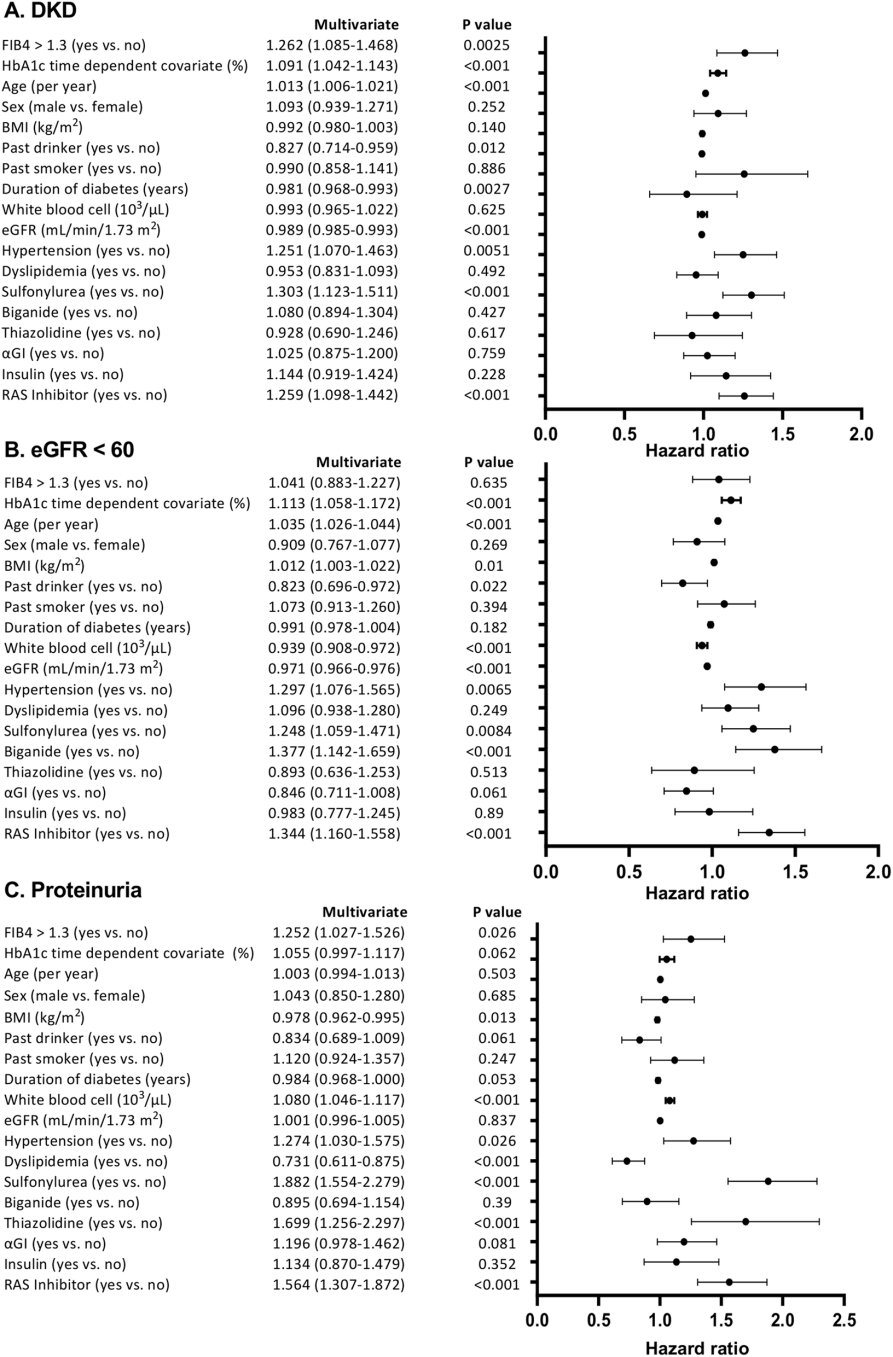
Cox proportional hazard ratios of FIB4 index > 1.3 for the development of (**A**) diabetic kidney disease (DKD: eGFR < 60 mL/min/1.73 m^2^ or proteinuria), (**B**) eGFR < 60 mL/min/1.73 m^2^, and (**C**) proteinuria in type 2 diabetic patients. Cox proportional hazard models were adjusted for age, sex, BMI, a HbA1c time dependent covariate, baseline eGFR, smoking and drinking status (current or past), comorbidities (hypertension, dyslipidemia) and anti-diabetic and anti-hypertensive medications. *95% CI* 95% confidence interval. N = 312.

**Figure 5:**
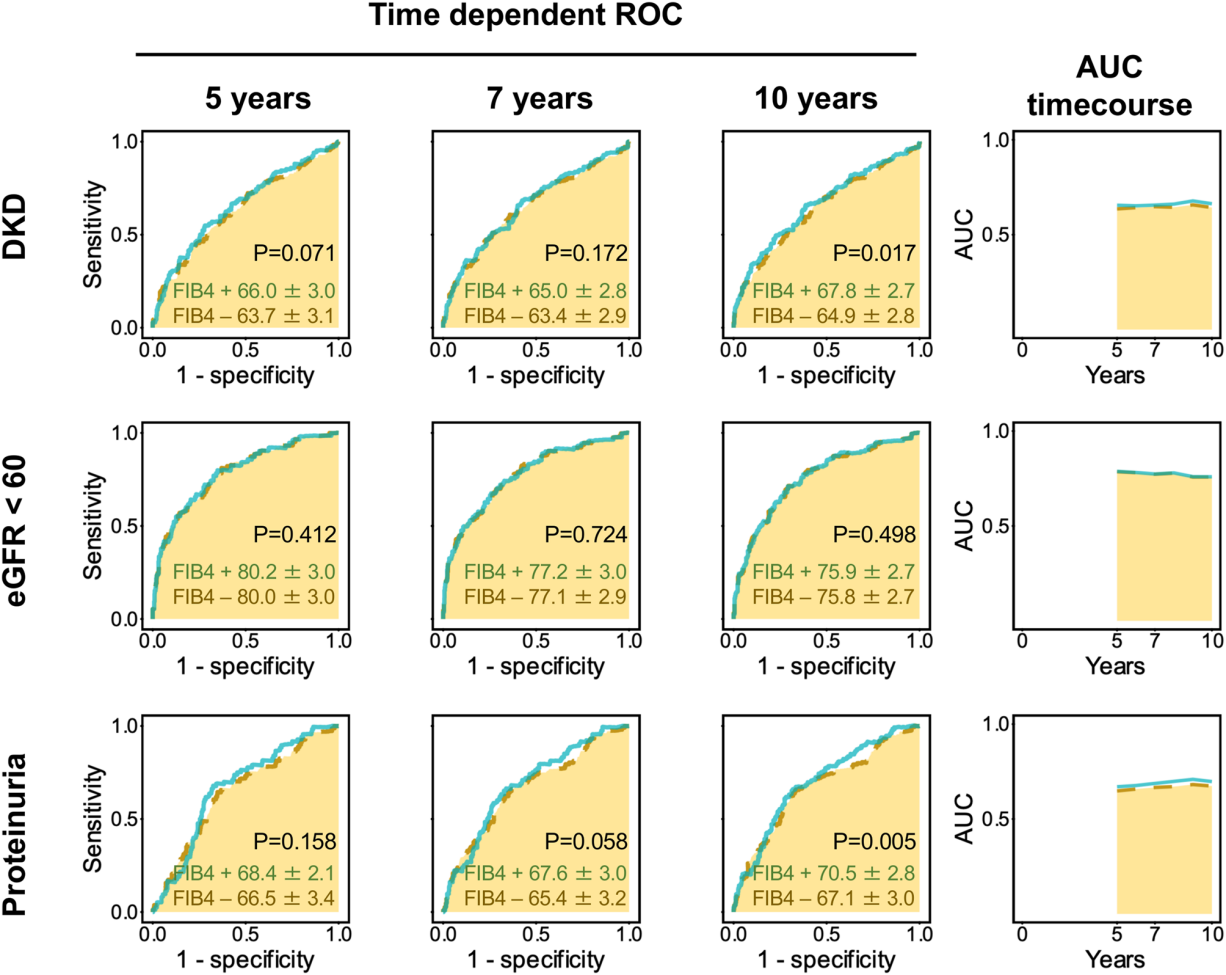
Time dependent receiver operating characteristic curve (ROC) analysis of FIB4 index for development of DKD, eGFR and proteinuria. Models included with or without FIB4 index were anlyzed by area under the curve (AUC) of ROC analysis. Model covariants except FIB4 index included age, sex, BMI, baseline HbA1c, baseline eGFR, smoking and drinking status (current or past), comorbidities (hypertension, dyslipidemia) and anti-diabetic and anti-hypertensive medications. P values were for comparisons between model with or without FIB4 index.

For sensitivity analyses: univariate and Cox proportional hazards models were repeated: (1) by using the FIB4 index as continuous (Supplementary Fig. 2) or quartile variables (Supplementary Fig. 3); (2) by HbA1c as a time dependent covariate plus possible emerging biomarker for DKD (white blood cell count) (Fig. 4); (3) by a new data-set with multiple imputation method for missing data analysis (Supplementary Figs. 4, 5): (4) time dependent ROC of FIB4 index for development of DKD, eGFR and proteinuria (Fig. 5).

In univariate and Cox proportional hazards models, (Supplementary Fig. 2) or quartile variables (Supplementary Fig. 3) the FIB4 index was a significant variable for the onset of DKD and proteinuria. When HbA1c during follow-up added as a time dependent covariate, FIB4 index > 1.3 was still a significant variable for the onset of DKD and proteinuria. Missing pattern of variables was shown in Supplementary Fig. 4. Duration of diabetes was frequent and missed mostly in one center (Tomishiro Central Hospital). The other variables were considered to be Missing Completely at Random (MCAR)(Little’s test for MCAR, P = 1.000). We therefore used only Fukushima Medical University database in the model including diabetes duration (Fig. 4). On a new data-set with multiple imputation method for missing data analysis, FIB4 index was also a significant variable for the onset of DKD and proteinuria. (Supplementary Fig. 5). Finally, we performed time dependent receiver operating characteristic curve (ROC) analysis of FIB4 index for development of DKD, eGFR and proteinuria (Fig. 5). The addition of FIB4 index in the classical risk model for the development of DKD and proteinuria significantly improved at 10 years, but not at 5 and 7 years. Annual changes in FIB4 index during the observational periods were shown in Supplementary Fig. 6. The values of FIB4 index gradually increased in the ≤ 1.3 and > 1.3 groups during over 10 years, but the value range of 25% to 75% ranges did not cross between groups (Supplementary Fig. 6). E-values, relative risk + √(relative risk (relative risk − 1), for DKD and proteinuria were 1.89 and 1.83, respectively, indicating unmeasured confounding variables with hazard ratios over these values may affect the impact of FIB4 index.

## Discussion

In this study, we investigated the impact of the FIB4 index > 1.3 on the development of DKD in Japanese type 2 diabetic patients and obtained two major findings. First, the group with the FIB4 index > 1.3 showed an increased DKD by Cox proportional HR and in the Kaplan–Meier curve. Second, the FIB4 index > 1.3 was associated with the development of proteinuria, but not with eGFR < 60. For the first time, this study demonstrated that the FIB4 index > 1.3, an index of liver fibrosis, has a prognostic impact on development of CKD, particularly on that of proteinuria, in Japanese type 2 diabetic patients.

Previous reports showed that the FIB4 index predicts onset of CKD in non-diabetic patients^22–25^. However, the prognostic impact of the FIB4 index in DKD remained unclarified. For the first time, this study exhibited that an FIB4 index > 1.3 was associated with onset of DKD in type 2 diabetic patients. Based on a study evaluating the utility of the FIB4 index as a marker of advanced fibrosis (bridging fibrosis or cirrhosis) in NAFLD, an FIB4 index ≥ 2.67 had an 80% positive predictive value and an FIB4 index ≤ 1.30 had a 90% negative predictive value^16^. Therefore, two groups, ≤ 1.3 and > 1.3 of FIB4 index, can be estimated as a group either excluded or not excluded for advanced liver fibrosis in type 2 diabetic patients. To elucidate which of liver fibrosis or fatty liver is crucial for DKD development, we evaluated the impact of ultrasonography-determined NAFLD for the DKD hazard ratio in type 2 diabetic patients in whom abdominal ultrasonography could be performed (n = 96). Multivariate analysis showed that the presence of NAFLD was not a significant predictor for onset of DKD. Onnerhag et al. reported that the stage of fibrosis, diagnosed by liver biopsy, was strongly correlated with the FIB4 index, and that the FIB4 index is a better predictor for metabolic complications, including CKD^18^. Collectively, the presence of liver fibrosis, but not the presence or absence of NAFLD, can be well correlated with the development of DKD. We calculated the optimal cutoff point of FIB4 index by the highest Youden index for developing DKD, eGFR < 60 mL/min/1.73 m^2^ and proteinuria (EZR 1.40)^26^ (Supplementary Table). Interestingly, the optimal cutoff points of FIB4 index for predicting development of DKD, eGFR < 60 and proteinuria were 1.296, 1.095 and 1.197, respectively, close to the cutoff (≤ 1.3) in the current study. This may support the notion that the exclusion of liver fibrosis is useful to predict delaying the onset of DKD.

In this study, an FIB4 index of > 1.3 was also correlated with the development of proteinuria, but not an eGFR of < 60. Yilmaz et al. reported that patients with microalbuminuria had a higher rate of fibrosis among non-diabetic patients with NAFLD^27^. Yeung et al. reported that liver fibrosis, but not liver steatosis, is associated with albuminuria in Chinese patients with type 2 diabetes^28^. These results agree with ours. On the other hand, our study showed no significant correlation between the FIB4 index and an eGFR < 60. In contrast, Onnerhag et al. reported a significant correlation between a high FIB4 index and the onset of eGFR < 60 in 144 Swedish NAFLD patients (included 22% diabetes mellitus)^18^. Although we cannot explain the discrepancy between our study and the one completed by Onnerhag et al., there might be different pathophysiological background between liver fibrosis and CKD with or without diabetes, as discussed below.

Two potential underlying mechanisms regarding why high values of the FIB4 index were linked to the development of DKD are discussed.

First, liver fibrosis, estimated by FIB4 index, might not be causal and could be a simple correlation to onset of CKD. Based on formula [age (year) × AST (IU/L)/(√ALT (IU/L) × platelet count (10^9^/L))]^29^, the FIB4 index can increase either by aging and an increase in AST to ALT ratio or by a decrease in platelet count. In patients with liver fibrosis, inhibition of thrombopoietin synthesis^30^, enhancement of platelets uptake by the liver with or without attendant splenomegaly can cause thrombocytopenia^31^. Currently, evidence lacks that thrombocytopenia is causally linked to the onset of CKD, at least an earlier stage of CKD^32^. In contrast, an increase in AST to ALT ratio could be elicited by metabolic derangements often observed in aging, diabetes mellitus/glucose intolerance/insulin resistance, and obesity. If so, FIB4 index may reflect a coincidental onset of liver fibrosis (NASH and LC)^29^ and CKD/DKD. Combined, we need to be careful to interpret whether the link between FIB4 index, including age, AST to ALT ratio and thrombocytopenia, and onset of CKD/DKD is a causal or a mere correlation relationship.

Second, an increase in the FIB4 index may be causally related to the onset of CKD/DKD. It is hypothesized that the progression of NAFLD into liver fibrosis (NASH) may be causally linked to the onset of CKD/DKD with four possible mechanisms: (1) A mechanism through the development of arteriosclerosis, in which NASH plays a crucial role by causing metabolic derangements such as dyslipidemia, insulin resistance, glucose intolerance, and dysproteinemia^33–36^, and those concomitantly enhance the RAS system and deactivates nitric oxide synthesis^37,38^. The proatherosclerotic state in liver fibrosis can facilitate the development of CKD/DKD. (2) A mechanism mediated by liver-derived inflammatory mediators and oxidative stress: In NASH, activation of the inflammation and production of reactive oxygen species enhance the release of proinflammatory, procoagulant, pro-oxidant, and profibrinogenic factors from the liver and those hepatokines may be involved in the development of CKD/DKD^4,11,39,40^. (3) A mechanism through hepatorenal syndrome (HRS): HRS is usually regarded as a detrimental condition in patients with end stage liver failure, such as LC^41^. However, this condition might be involved, at least partly, in the development of CKD/DKD: CKD/DKD may be resulting from a decrease in renal blood flow caused by a decrease in effective circulating blood volume due to whole body vasodilatation, increased portal pressure, and a decrease in cardiac output^41^. (4) A mechanism through insulin resistance (IR). Lipid accumulation in non-adipose tissues is called ectopic fat deposition^42^, which typically occurs in the liver of individuals with visceral fat obesity. Increased fatty acid fluxes from visceral fat cause hepatic insulin resistance^43^ which lead to simple hepatic steatosis. Vicious cycle of worsening insulin resistance in hepatic steatosis can promote the progression from simple fatty liver (NAFLD) to NASH possibly via a multifactorial process involving oxidative stress, lipid peroxidation, and mitochondrial dysfunction^44,45^. The net effect of hepatic insulin resistance elucidates insulin resistance in whole-body^44,45^ and may also be linked to that in the kidney^46^. Thus, insulin resistance in the liver and kidney might contribute synergistically to the progression of kidney disease by various mechanisms, including worsening diabetic control, activation of the sympathetic nervous system, sodium retention, and downregulation of the natriuretic peptide system^8,46^.

There are limitations in this study. First, since a liver biopsy was not performed, the correlation between the FIB4 index and the actual degree of fibrosis is not objective. Second, this was a retrospective cohort study and the causal or correlation relationship cannot be determined in this study. Third, this study comprised of only Japanese race from only two centers, suggesting a possibility of selection bias. Fourth, it could be arguable that respective assessment of “proteinuria” and “worsening eGFR” are clinically relevant or not^4^. Since progression of proteinuria is the main driver of the DKD, it might be meaningless to differentiate “proteinuria” and “worsening eGFR” separately.

Strength of this study. We performed several sensitivity analysis to validate our results. Cox proportional hazards models, by using continuous (Supplementary Fig. 2) and quartile variables (Supplementary Fig. 3), by time dependent covariate of follow-up HbA1c, and by multiple imputation method for missing data also found that the FIB4 index was a significant variable for the onset of DKD and proteinuria. Finally, time dependent ROC analysis of FIB4 index confirmed that this addition of FIB4 index may be useful during a longer period ~ 10 years.

## Conclusions

This study has demonstrated that the FIB4 index > 1.3, an index of liver fibrosis, has a prognostic impact on development of CKD/DKD and proteinuria in Japanese type 2 diabetic patients. Our results may warrant us usefulness of monitoring FIB4 index in diabetic patients and/or renal function in patients with a high FIB4 index. To conclude this, we need to confirm the prognostic impact of FIB4 index on the development of CKD/DKD or proteinuria in future studies.

## Methods

### Study design and subjects

This is an observational retrospective cohort study (STROBE Statement). The study protocol was approved by the Fukushima Medical University Ethics Committee (Number 29118) and the Tomishiro Central Hospital Ethics Committee (R01R027). This study was conducted according also to the Ethical Guidelines for Medical and Health Research Involving Human Subjects enacted by MHLW of Japan [http://www.mhlw.go.jp/file/06-Seisakujouhou-10600000-Daijinkanboukouseikagakuka/0000069410.pdf and http://www.mhlw.go.jp/file/06-Seisakujouhou-10600000-Daijinkanboukouseikagakuka/0000080278.pdf]. Inclusion criteria of the participants was adult patients with type 2 diabetes mellitus who had visited the Department of Diabetes, Endocrinology, and Metabolism, Fukushima Medical University Hospital or Department of Diabetes and Lifestyle-Related Disease Center, Tomishiro Central Hospital between January 2002 and March 2019. Written informed consent was obtained from the patients between January 2018 and March 2019 in the Department of Diabetes, Endocrinology, and Metabolism, School of Medicine, Fukushima Medical University Hospital and informed consent for participants in Tomishiro Central Hospital was waived by the Tomishiro Central Hospital Ethics Committee. Instead, we publicized information concerning this study in the Hospital and ensured that the subjects could refuse the use of their personal information. Total of 1197 patients with type 2 diabetes mellitus were selected from both hospitals on their medical records (Fig. 1). On the below definition of DKD, 279 CKD/DKD at baseline were excluded. On exclusion criteria, 81 were removed by complications of liver, kidney and hematologic diseases (Fig. 1). Twenty-four patients with non-diabetic kidney diseases (chronic glomerulonephritis, vasculitis, polycystic kidney disease, and renal cancer) and 47 patients with liver disease other than NAFLD (viral hepatitis, autoimmune liver disease, liver transplantation). Patients diagnosed with liver cirrhosis and heavy drinker (consumption of ethanol less than 20 g/day for women and 30 g/day for men) had been excluded in advance. After deleted for 146 with observation period < 1 year and 107 with missing data, the remaining 584 patients with type 2 diabetes mellitus were enrolled and their paper and/or electrical medical records were scrutinized from October 2002 to March 2019. Their first visit to either hospitals was considered as the baseline. The parameters such as age, sex, history of diabetes, family and social history, medical checkup history, complications, medications, laboratory data, and all dates were recorded.

### Biochemical measurements

Laboratory parameters were measured by standard assays. In brief, HbA1c was measured by ion exchange high performance liquid chromatography in automated glycohemoglobin analyzer (HLC-723G8/G9, Tosoh Corp., Tokyo, Japan). Creatinine was measured by an enzymatic assay in clinical chemistry analyzer (ARCHITECT c16000, Abbott, Illinois, USA). Semiqualitative proteinuria was assessed by urinary dipstick test.

### Ultrasonography

Standard abdominal ultrasonography was performed in a part of patients after overnight fasting. Diagnosis of NAFLD was based on the increased echogenicity of the liver parenchyma as compared to the right kidney’s cortex. Visibility and sharpness of the diaphragm and hepatic veins’ interface were analyzed as well. Based on these 3 parameters was further classified into 3 grades: Grade 0, no steatosis; Grade 1, mild steatosis; Grade 2, moderate steatosis; Grade 3, severe steatosis as described^47^.

### Definition

Diabetes mellitus was defined by a fasting plasma glucose ≥ 126 mg/dL, random plasma glucose ≥ 200 mg/dL, and/or HbA1c ≥ 6.5% (48 mmol/mol) or use of anti-diabetic medication [World Health Organization (2006) Definition and Diagnosis of Diabetes Mellitus and Intermediate Hyperglycemia: Report of a WHO/IDF Consultation. https://www.who.int/diabetes/publications/diagnosis_diabetes2006/en/]. Patients with type 1 diabetes mellitus or secondary diabetes were excluded. The FIB4 index was calculated by Age (year) × AST (IU/L)/(√ALT (IU/L) × Platelet count (10^9^/L))^29,48^. A cut-off value of 1.3 or less, which was 90% negative for the progression of liver fibrosis, was applied^16^. The definition of DKD was eGFR < 60 mL/min/1.73 m^2^ and/or proteinuria 1 + with urinary dipsctick test. The primary endpoint of this study was onset of DKD. The secondary endpoint of this study was each onset of eGFR < 60 mL/min/1.73 m^2^ or proteinuria 1 + with a dipstick urine test. We calculated eGFR using the Japanese formula for GFR estimation, i.e., eGFR (mL/min/1.73 m^2^) = 194 × serum creatinine (mg/dL)^−1.094^ × age (years)^−0.287^^49^. Hypertension was defined as systolic blood pressure ≥ 140 mmHg or diastolic blood pressure ≥ 90 mmHg or those taking antihypertensive drugs. Dyslipidemia was defined as LDL cholesterol ≥ 140 mg/dL or those taking antihyperlipidemic drugs.

### Statistical analysis

Continuous and parametric values are expressed as mean ± standard deviation, and the nonparametric variables as median (first quartile-third quartile). The two-tailed unpaired student's t-test and Mann–Whitney *U* test were used for parametric and non-parametric data, respectively. Categorical variables are shown as percentages and were analyzed using the eχ^2 ^test. Univariate survival analysis was calculated using the Kaplan–Meier curve and analyzed by a log rank test. Univariate and Cox proportional hazards model were used to determine the independent contributions of the FIB4 index as a dichotomizing variable (> 1.3 vs. ≤ 1.3) to the development of DKD, a decline in eGFR (< 60 mL/min/1.73 m^2^), or proteinuria after adjusting for age, sex, BMI, baseline HbA1c, baseline eGFR, smoking and drinking status (current or past), comorbidities (hypertension, dyslipidemia) and anti-diabetic and anti-hypertensive medications. Covariates used for the Cox proportional hazards model were chosen from possible confounding factors for DKD^1–4^.

For sensitivity analyses: univariate and Cox proportional hazards models were repeated: (1) by using the FIB4 index as continuous or quartile variable≈; (2) by HbA1c as a time dependent covariate plus possible emerging biomarker for DKD (white blood cell count); (3) by a new data-set with multiple imputation method for missing data analysis: (4) time dependent AUC of FIB4 index for the develeopme of DKD, eGFR < 60 and proteinuria.

Values of *P* < 0.05 were considered as statistically significant. Statistical analyses were conducted using SPSS version 25 (SPSS, Inc., Chicago, Illinois, USA) or R 3.6.3. VIM package 5.1.1 and ggplot2 3.3 running on R 3.6.3 are used for visualization of the missing pattern.

### Ethics approval and consent to participate

The study protocol was approved by the Fukushima Medical University Ethics Committee (Number 29118) and the Tomishiro Central Hospital Ethics Committee (R01R027). Written informed consent was obtained from the patients recruited between January 2018 and March 2019 in the Department of Diabetes, Endocrinology, and Metabolism, School of Medicine, Fukushima Medical University Hospital. Informed consent for participants in Tomishiro Central Hospital was waived by the Tomishiro Central Hospital Ethics Committee. Instead, we publicized information concerning this study in the Hospital and ensured that the subjects could refuse the use of their personal information.

## Supplementary Information

Supplementary Figure 1.

Supplementary Figure 2.

Supplementary Figure 3.

Supplementary Figure 4.

Supplementary Figure 5.

Supplementary Figure 6.

Supplementary Table.

Supplementary Information.

## Data Availability

All data generated or analyzed during this study are included in this published article.
