# Supplementary material for: High FIB4 index is an independent risk factor of diabetic kidney disease in type 2 diabetes
Source: Sci Rep. 2021 Jun 3;11:11753. doi: 10.1038/s41598-021-88285-6 (PMC8175689; doi:10.1038/s41598-021-88285-6)
Supplement: Supplementary file 1 — Supplementary Figure 1. [file 41598_2021_88285_MOESM1_ESM.pdf]

High FIB4 index is an independent risk factor of diabetic kidney disease in type 2 diabetes

Haruka Saito[1], Hayato Tanabe[1], Akihiro Kudo[1], Noritaka Machii[1], Moritake Higa[2], Satoshi Yamaguchi[1,5], Gulinu Maimaituxun[1], Kazumichi Abe[3], Atsushi Takahashi[3], Kenichi Tanaka[4], Koichi Asahi[6], Hiroaki Masuzaki[7], Hiromasa Ohira[3], Junichiro J. Kazama[4] and Michio Shimabukuro[1*]

[1]Department of Diabetes, Endocrinology and Metabolism, [3]Department of Gastroenterology and [4]Department of Nephrology and Hypertension, Fukushima Medical University, Fukushima, Japan; [2]Department of Diabetes and Lifestyle-Related Disease Center, Tomishiro Central Hospital, Okinawa, Japan; [5]Department of Cardiology, Nakagami Hospital, Okinawa, Japan; [6]Division of Nephrology and Hypertension, Iwate Medical University, Iwate, Japan; [7]Division of Endocrinology, Diabetes and Metabolism, Hematology, Rheumatology (Second Department of Internal Medicine), University of the Ryukyu, Okinawa, Japan.

*Corresponding author at: Department of Diabetes, Endocrinology and Metabolism, Fukushima Medical University, 1 Hikarigaoka, Fukushima City, Fukushima 960-1295, Japan; E-mail address: mshimabukuro-ur@umin.ac.jp (M. Shimabukuro); Tel +81-24-547-1305 , Fax +81-24-547-1311

	Univariate	P value	Multivariate	P value
FIB4 > 1.3 (yes vs. no)	2.00 (1.45-2.76)	<0.01	1.72 (1.16-2.56)	<0.01
Age (years)	1.02 (1.01-1.04)	<0.01	1.01 (0.99-1.03)	0.53
Sex (male vs. female)	1.13 (0.84-1.52)	0.42	1.06 (0.71-1.59)	0.77
BMI (kg/m^2)	0.99 (0.97-1.02)	0.57	1.00 (0.98-1.03)	0.79
eGFR (mL/min/1.73 m^2)	0.98 (0.97-0.99)	<0.01	0.99 (0.98-0.99)	<0.05
Median HbA1c (%)	0.97 (0.81-1.17)	0.78	1.06 (0.85-1.32)	0.62
Hypertension (yes vs. no)	1.28 (0.91-1.80)	0.16	1.03 (0.69-1.55)	0.89
Dyslipidemia (yes vs. no)	0.96 (0.69-1.34)	0.81	0.93 (0.66-1.32)	0.68
Past drinker (yes vs. no)	1.00 (0.74-1.34)	0.98	0.90 (0.62-1.31)	0.57
Past smoker (yes vs. no)	1.06 (0.79-1.42)	0.72	1.07 (0.74-1.54)	0.72
Sulfonylurea (yes vs. no)	1.22 (0.88-1.69)	0.23	1.08 (0.73-1.60)	0.71
Biguanide (yes vs. no)	1.17 (0.78-1.75)	0.46	1.13 (0.73-1.74)	0.58
Thiazolidine (yes vs. no)	1.33 (0.70-2.52)	0.39	1.36 (0.70-2.64)	0.36
αGI (yes vs. no)	0.99 (0.68-1.44)	0.95	0.83 (0.54-1.25)	0.37
Insulin (yes vs. no)	1.68 (1.10-2.58)	<0.05	1.49 (0.94-2.36)	0.09
RAS inhibitor (yes vs. no)	1.53 (1.12-2.08)	<0.01	1.35 (0.95-1.91)	0.10

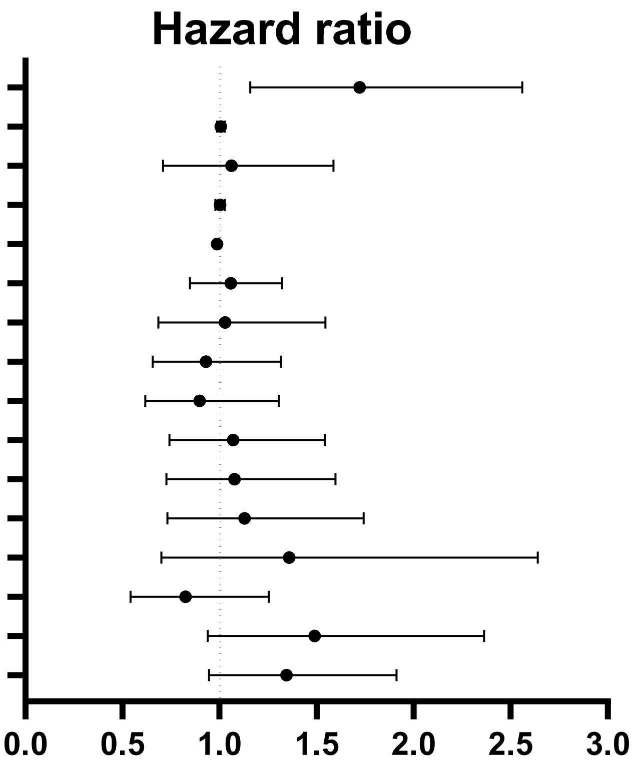

Supplement Figure 1. Hazard ratios for the development of diabetic kidney disease (DKD: eGFR < 60 mL/min/1.73 m^2 or proteinuria in type 2 diabetic patients (Cox proportional hazards model). 95% CI, 95% confidence interval. In this model, median HbA1c (%) was used inplace of baseline HbA1c in **Figure 3A**.
