# Supplementary material for: High FIB4 index is an independent risk factor of diabetic kidney disease in type 2 diabetes
Source: Sci Rep. 2021 Jun 3;11:11753. doi: 10.1038/s41598-021-88285-6 (PMC8175689; doi:10.1038/s41598-021-88285-6)
Supplement: Supplementary file 2 — Supplementary Figure 2. [file 41598_2021_88285_MOESM2_ESM.pdf]

High FIB4 index is an independent risk factor of diabetic kidney disease in type 2 diabetes

Haruka Saito[1], Hayato Tanabe[1], Akihiro Kudo[1], Noritaka Machii[1], Moritake Higa[2], Satoshi Yamaguchi[1,5], Gulinu Maimaituxun[1], Kazumichi Abe[3], Atsushi Takahashi[3], Kenichi Tanaka[4], Koichi Asahi[6], Hiroaki Masuzaki[7], Hiromasa Ohira[3], Junichiro J. Kazama[4] and Michio Shimabukuro[1*]

[1]Department of Diabetes, Endocrinology and Metabolism, [3]Department of Gastroenterology and [4]Department of Nephrology and Hypertension, Fukushima Medical University, Fukushima, Japan; [2]Department of Diabetes and Lifestyle-Related Disease Center, Tomishiro Central Hospital, Okinawa, Japan; [5]Department of Cardiology, Nakagami Hospital, Okinawa, Japan; [6]Division of Nephrology and Hypertension, Iwate Medical University, Iwate, Japan; [7]Division of Endocrinology, Diabetes and Metabolism, Hematology, Rheumatology (Second Department of Internal Medicine), University of the Ryukyu, Okinawa, Japan.

*Corresponding author at: Department of Diabetes, Endocrinology and Metabolism, Fukushima Medical University, 1 Hikarigaoka, Fukushima City, Fukushima 960-1295, Japan; E-mail address: mshimabukuro-ur@umin.ac.jp (M. Shimabukuro); Tel +81-24-547-1305 , Fax +81-24-547-1311

A. DKD

	Univariate	P value	Multivariate	P value
FIB4 Index (per 1 unit)	1.254 (1.115-1.410)	<0.001	1.285 (1.107-1.492)	0.001
Age (per year)	1.019 (1.007-1.032)	0.001	1.005 (0.990-1.019)	0.522
Sex (male vs. female)	1.196 (0.939-1.523)	0.147	1.130 (0.816-1.565)	0.460
BMI (kg/m^2)	1.001 (0.981-1.022)	0.897	1.008 (0.988-1.028)	0.433
eGFR (mL/min/1.73 m^2)	0.989 (0.981-0.997)	0.008	0.988 (0.979-0.996)	0.005
HbA1c (%)	1.053 (0.998-1.111)	0.062	1.066 (1.004-1.133)	0.038
Hypertension (yes vs. no)	1.473 (1.146-1.893)	0.002	1.264 (0.955-1.673)	0.101
Dyslipidemia (yes vs. no)	1.015 (0.779-1.322)	0.914	0.941 (0.713-1.241)	0.665
Past drinker (yes vs. no)	1.003 (0.794-1.266)	0.982	0.917 (0.680-1.239)	0.574
Past smoker (yes vs. no)	1.221 (0.967-1.541)	0.094	1.244 (0.942-1.643)	0.124
Sulfonylurea (yes vs. no)	1.524 (1.150-2.020)	0.003	1.411 (1.016-1.959)	0.040
Biguanide (yes vs. no)	1.188 (0.847-1.667)	0.318	1.088 (0.759-1.558)	0.646
Thiazolidine (yes vs. no)	1.110 (0.621-1.981)	0.725	1.005 (0.557-1.812)	0.987
αGI (yes vs. no)	1.093 (0.760-1.571)	0.633	0.844 (0.566-1.259)	0.844
Insulin (yes vs. no)	1.720 (1.130-2.616)	0.011	1.470 (0.936-2.311)	0.095
RAS inhibitor (yes vs. no)	1.616 (1.236-2.112)	<0.001	1.431 (1.060-1.932)	0.019

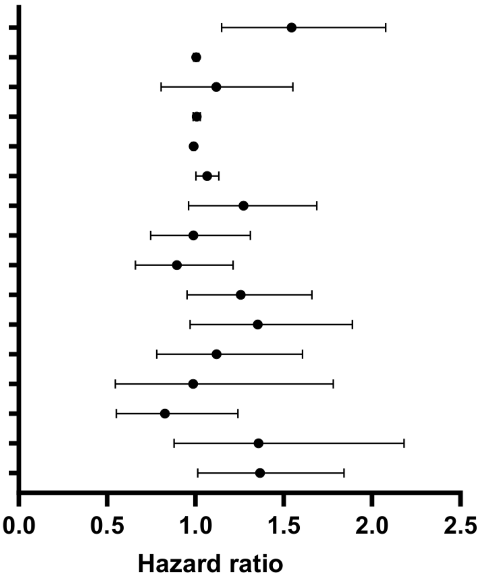

B. eGFR < 60

	Univariate	P value	Multivariate	P value
FIB4 Index (per 1 unit)	1.314 (1.142-1.511)	<0.001	1.220 (0.982-1.518)	0.073
Age (per year)	1.055 (1.039-1.071)	<0.001	1.032 (1.012-1.052)	0.002
Sex (male vs. female)	0.809 (0.604-1.083)	0.155	0.842 (0.558-1.269)	0.411
BMI (kg/m^2)	0.999 (0.972-1.026)	0.922	1.019 (0.995-1.044)	0.122
eGFR (mL/min/1.73 m^2)	0.953 (0.940-0.965)	<0.001	0.957 (0.944-0.970)	<0.001
HbA1c (%)	1.060 (0.993-1.132)	0.081	1.111 (1.030-1.198)	0.007
Hypertension (yes vs. no)	1.864 (1.338-2.598)	<0.001	1.370 (0.946-1.985)	0.096
Dyslipidemia (yes vs. no)	1.097 (0.784-1.536)	0.589	1.104 (0.776-1.572)	0.581
Past drinker (yes vs. no)	0.691 (0.517-0.925)	0.013	0.867 (0.590-1.274)	0.467
Past smoker (yes vs. no)	0.851 (0.636-1.139)	0.278	1.109 (0.771-1.594)	0.578
Sulfonylurea (yes vs. no)	1.992 (1.433-2.769)	<0.001	1.522 (1.030-2.250)	0.035
Biguanide (yes vs. no)	1.639 (1.124-2.389)	0.010	1.399 (0.930-2.103)	0.107
Thiazolidine (yes vs. no)	1.197 (0.588-2.435)	0.620	1.049 (0.509-2.163)	0.896
αGI (yes vs. no)	1.305 (0.850-2.005)	0.224	0.867 (0.542-1.388)	0.552
Insulin (yes vs. no)	1.735 (1.052-2.860)	0.031	1.235 (0.714-2.138)	0.450
RAS inhibitor (yes vs. no)	1.952 (1.429-2.666)	<0.001	1.443 (1.013-2.055)	0.042

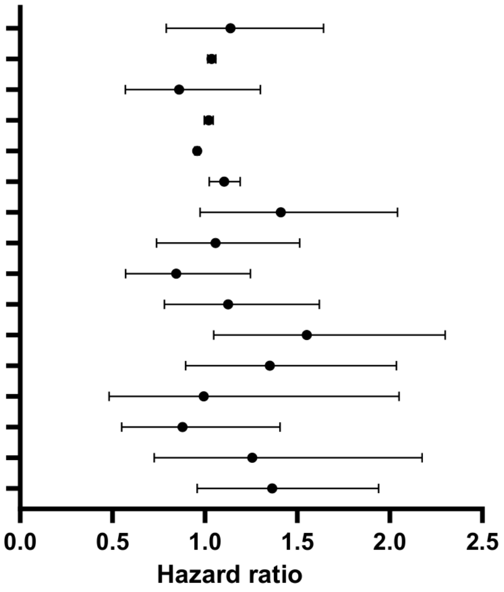

C. Proteinuria

	Univariate	P value	Multivariate	P value
FIB4 Index (per 1 unit)	1.195 (1.007-1.417)	0.041	1.261 (1.051-1.513)	0.012
Age (per year)	0.995 (0.980-1.010)	0.518	0.992 (0.974-1.010)	0.391
Sex (male vs. female)	1.644 (1.191-2.267)	0.002	1.316 (0.865-2.001)	0.199
BMI (kg/m^2)	1.007 (0.982-1.033)	0.561	1.008 (0.983-1.033)	0.543
eGFR (mL/min/1.73 m^2)	1.008 (1.001-1.015)	0.026	1.004 (0.996-1.013)	0.329
HbA1c (%)	1.088 (1.018-1.163)	0.013	1.071 (0.991-1.157)	0.082
Hypertension (yes vs. no)	1.392 (1.012-1.913)	0.042	1.205 (0.849-1.711)	0.296
Dyslipidemia (yes vs. no)	0.986 (0.708-1.373)	0.931	0.908 (0.641-1.286)	0.587
Past drinker (yes vs. no)	1.286 (0.955-1.731)	0.098	0.988 (0.687-1.422)	0.949
Past smoker (yes vs. no)	1.739 (1.290-2.345)	<0.001	1.470 (1.031-2.096)	0.033
Sulfonylurea (yes vs. no)	1.563 (1.108-2.204)	0.011	1.420 (0.955-2.111)	0.083
Biguanide (yes vs. no)	1.169 (0.770-1.772)	0.464	1.091 (0.700-1.700)	0.701
Thiazolidine (yes vs. no)	1.749 (0.948-3.226)	0.073	1.790 (0.958-3.346)	0.068
αGI (yes vs. no)	1.089 (0.696-1.706)	0.709	0.845 (0.517-1.381)	0.501
Insulin (yes vs. no)	1.514 (0.892-2.570)	0.125	1.297 (0.732-2.295)	0.373
RAS inhibitor (yes vs. no)	1.567 (1.123-2.189)	0.008	1.407 (0.964-2.053)	0.077

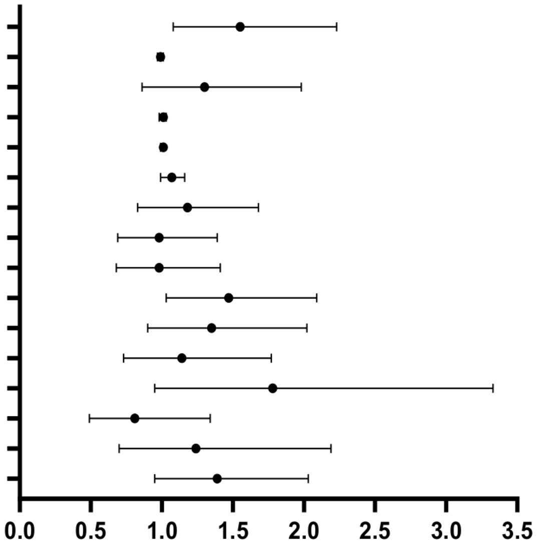

Supplement Figure 2. Univariate and Cox proportional hazard ratios of FIB4 index (per 1 unit) for the development of (A) diabetic kidney disease (DKD: eGFR < 60 mL/min/1.73 m2 or proteinuria), (B) eGFR < 60 mL/min/1.73 m2, and (C) proteinuria in type 2 diabetic patients. Cox proportional hazard models were adjusted for age, sex, BMI, baseline HbA1c, baseline eGFR, smoking and drinking status (current or past), comorbidities (hypertension, dyslipidemia) and anti-diabetic and anti-hypertensive medications. 95% CI, 95% confidence interval.
