# Supplementary material for: High FIB4 index is an independent risk factor of diabetic kidney disease in type 2 diabetes
Source: Sci Rep. 2021 Jun 3;11:11753. doi: 10.1038/s41598-021-88285-6 (PMC8175689; doi:10.1038/s41598-021-88285-6)
Supplement: Supplementary file 3 — Supplementary Figure 3. [file 41598_2021_88285_MOESM3_ESM.pdf]

High FIB4 index is an independent risk factor of diabetic kidney disease in type 2 diabetes

Haruka Saito[1], Hayato Tanabe[1], Akihiro Kudo[1], Noritaka Machii[1], Moritake Higa[2], Satoshi Yamaguchi[1,5], Gulinu Maimaituxun[1], Kazumichi Abe[3], Atsushi Takahashi[3], Kenichi Tanaka[4], Koichi Asahi[6], Hiroaki Masuzaki[7], Hiromasa Ohira[3], Junichiro J. Kazama[4] and Michio Shimabukuro[1*]

[1]Department of Diabetes, Endocrinology and Metabolism, [3]Department of Gastroenterology and [4]Department of Nephrology and Hypertension, Fukushima Medical University, Fukushima, Japan; [2]Department of Diabetes and Lifestyle-Related Disease Center, Tomishiro Central Hospital, Okinawa, Japan; [5]Department of Cardiology, Nakagami Hospital, Okinawa, Japan; [6]Division of Nephrology and Hypertension, Iwate Medical University, Iwate, Japan; [7]Division of Endocrinology, Diabetes and Metabolism, Hematology, Rheumatology (Second Department of Internal Medicine), University of the Ryukyu, Okinawa, Japan.

*Corresponding author at: Department of Diabetes, Endocrinology and Metabolism, Fukushima Medical University, 1 Hikarigaoka, Fukushima City, Fukushima 960-1295, Japan; E-mail address: mshimabukuro-ur@umin.ac.jp (M. Shimabukuro); Tel +81-24-547-1305 , Fax +81-24-547-1311

A. DKD

	Multivariate	P value
FIB4 Quartile	1.202(1.051-1.374)	0.007
Age (per year)	1.003(0.988-1.018)	0.707
Sex (male vs. female)	1.114(0.805-1.542)	0.514
BMI (kg/m2)	1.008(0.989-1.028)	0.407
eGFR (mL/min/1.73 m2)	0.990(0.981-0.998)	0.017
HbA1c (%)	1.066(1.003-1.132)	0.039
Hypertension (yes vs. no)	1.273(0.962-1.686)	0.092
Dyslipidemia (yes vs. no)	0.969(0.732-1.281)	0.824
Past drinker (yes vs. no)	0.905(0.67-1.224)	0.518
Past smoker (yes vs. no)	1.256(0.952-1.658)	0.107
Sulfonylurea (yes vs. no)	1.395(1.004-1.939)	0.047
Biganide (yes vs. no)	1.086(0.758-1.557)	0.653
Thiazolidine (yes vs. no)	0.978(0.543-1.762)	0.94
αGI (yes vs. no)	0.847(0.568-1.264)	0.416
Insulin (yes vs. no)	1.42(0.901-2.237)	0.13
RAS Inhibitor (yes vs. no)	1.40(1.039-1.887)	0.027

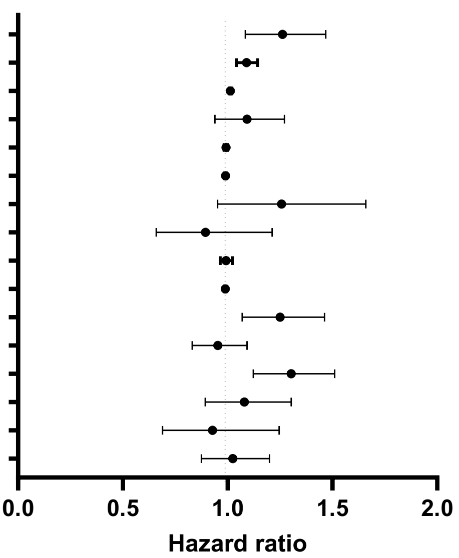

B. eGFR < 60

	Multivariate	P value
FIB4 Quartile	1.109(0.937-1.314)	0.230
Age (per year)	1.033(1.012-1.054)	0.002
Sex (male vs. female)	0.834(0.553-1.259)	0.388
BMI (kg/m2)	1.019(0.996-1.044)	0.112
eGFR (mL/min/1.73 m2)	0.958(0.945-0.971)	<0.001
HbA1c (%)	1.109(1.028-1.196)	0.007
Hypertension (yes vs. no)	1.399(0.966-2.025)	0.075
Dyslipidemia (yes vs. no)	1.092(0.767-1.555)	0.625
Past drinker (yes vs. no)	0.859(0.583-1.267)	0.444
Past smoker (yes vs. no)	1.117(0.776-1.609)	0.552
Sulfonylurea (yes vs. no)	1.522(1.028-2.252)	0.036
Biganide (yes vs. no)	1.390(0.922-2.094)	0.116
Thiazolidine (yes vs. no)	1.013(0.492-2.086)	0.971
αGI (yes vs. no)	0.845(0.528-1.352)	0.482
Insulin (yes vs. no)	1.233(0.71-2.142)	0.457
RAS Inhibitor (yes vs. no)	1.402(0.987-1.991)	0.059

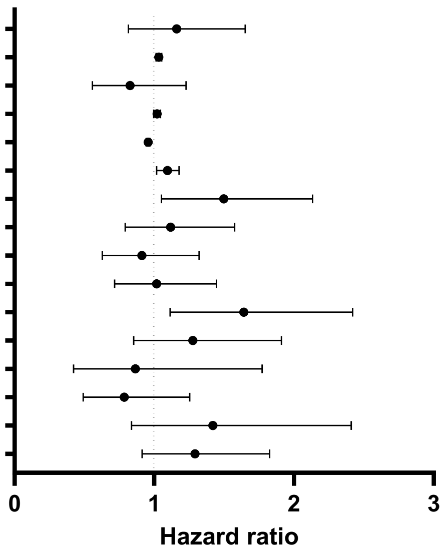

C. Proteinuria

	Multivariate	P value
FIB4 Quartile	1.246(1.058-1.469)	0.009
Age (per year)	0.988(0.969-1.007)	0.207
Sex (male vs. female)	1.299(0.857-1.969)	0.218
BMI (kg/m2)	1.008(0.983-1.034)	0.518
eGFR (mL/min/1.73 m2)	1.005(0.997-1.014)	0.201
HbA1c (%)	1.071(0.992-1.156)	0.081
Hypertension (yes vs. no)	1.176(0.827-1.672)	0.366
Dyslipidemia (yes vs. no)	0.958(0.675-1.36)	0.81
Past drinker (yes vs. no)	0.971(0.674-1.397)	0.873
Past smoker (yes vs. no)	1.493(1.047-2.127)	0.027
Sulfonylurea (yes vs. no)	1.400(0.943-2.08)	0.095
Biganide (yes vs. no)	1.112(0.713-1.736)	0.639
Thiazolidine (yes vs. no)	1.786(0.957-3.333)	0.069
αGI (yes vs. no)	0.868(0.531-1.42)	0.573
Insulin (yes vs. no)	1.251(0.706-2.219)	0.443
RAS Inhibitor (yes vs. no)	1.433(0.98-2.095)	0.063

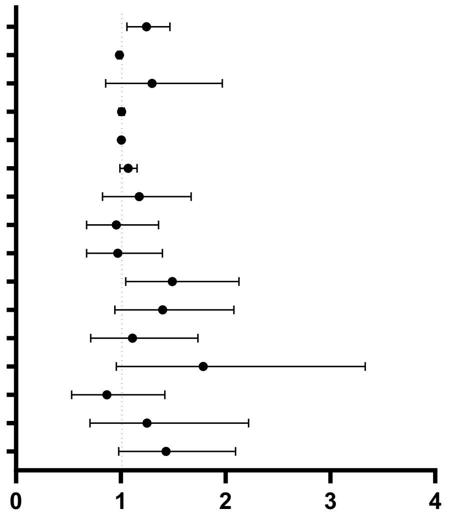

Supplement Figure 3. Univariate and Cox proportional hazard ratios of FIB4 quartile for the development of (A) diabetic kidney disease (DKD: eGFR < 60 mL/min/1.73 m2 or proteinuria), (B) eGFR < 60 mL/min/1.73 m2, and (C) proteinuria in type 2 diabetic patients. Cox proportional hazard models were adjusted for age, sex, BMI, baseline HbA1c, baseline eGFR, smoking and drinking status (current or past), comorbidities (hypertension, dyslipidemia) and anti-diabetic and anti-hypertensive medications. 95% CI, 95% confidence interval.
