# Supplementary material for: High FIB4 index is an independent risk factor of diabetic kidney disease in type 2 diabetes
Source: Sci Rep. 2021 Jun 3;11:11753. doi: 10.1038/s41598-021-88285-6 (PMC8175689; doi:10.1038/s41598-021-88285-6)
Supplement: Supplementary file 4 — Supplementary Figure 4. [file 41598_2021_88285_MOESM4_ESM.pdf]

High FIB4 index is an independent risk factor of diabetic kidney disease in type 2 diabetes

Haruka Saito[1], Hayato Tanabe[1], Akihiro Kudo[1], Noritaka Machii[1], Moritake Higa[2], Satoshi Yamaguchi[1,5], Gulinu Maimaituxun[1], Kazumichi Abe[3], Atsushi Takahashi[3], Kenichi Tanaka[4], Koichi Asahi[6], Hiroaki Masuzaki[7], Hiromasa Ohira[3], Junichiro J. Kazama[4] and Michio Shimabukuro[1*]

[1]Department of Diabetes, Endocrinology and Metabolism, [3]Department of Gastroenterology and [4]Department of Nephrology and Hypertension, Fukushima Medical University, Fukushima, Japan; [2]Department of Diabetes and Lifestyle-Related Disease Center, Tomishiro Central Hospital, Okinawa, Japan; [5]Department of Cardiology, Nakagami Hospital, Okinawa, Japan; [6]Division of Nephrology and Hypertension, Iwate Medical University, Iwate, Japan; [7]Division of Endocrinology, Diabetes and Metabolism, Hematology, Rheumatology (Second Department of Internal Medicine), University of the Ryukyu, Okinawa, Japan.

*Corresponding author at: Department of Diabetes, Endocrinology and Metabolism, Fukushima Medical University, 1 Hikarigaoka, Fukushima City, Fukushima 960-1295, Japan; E-mail address: mshimabukuro-ur@umin.ac.jp (M. Shimabukuro); Tel +81-24-547-1305 , Fax +81-24-547-1311

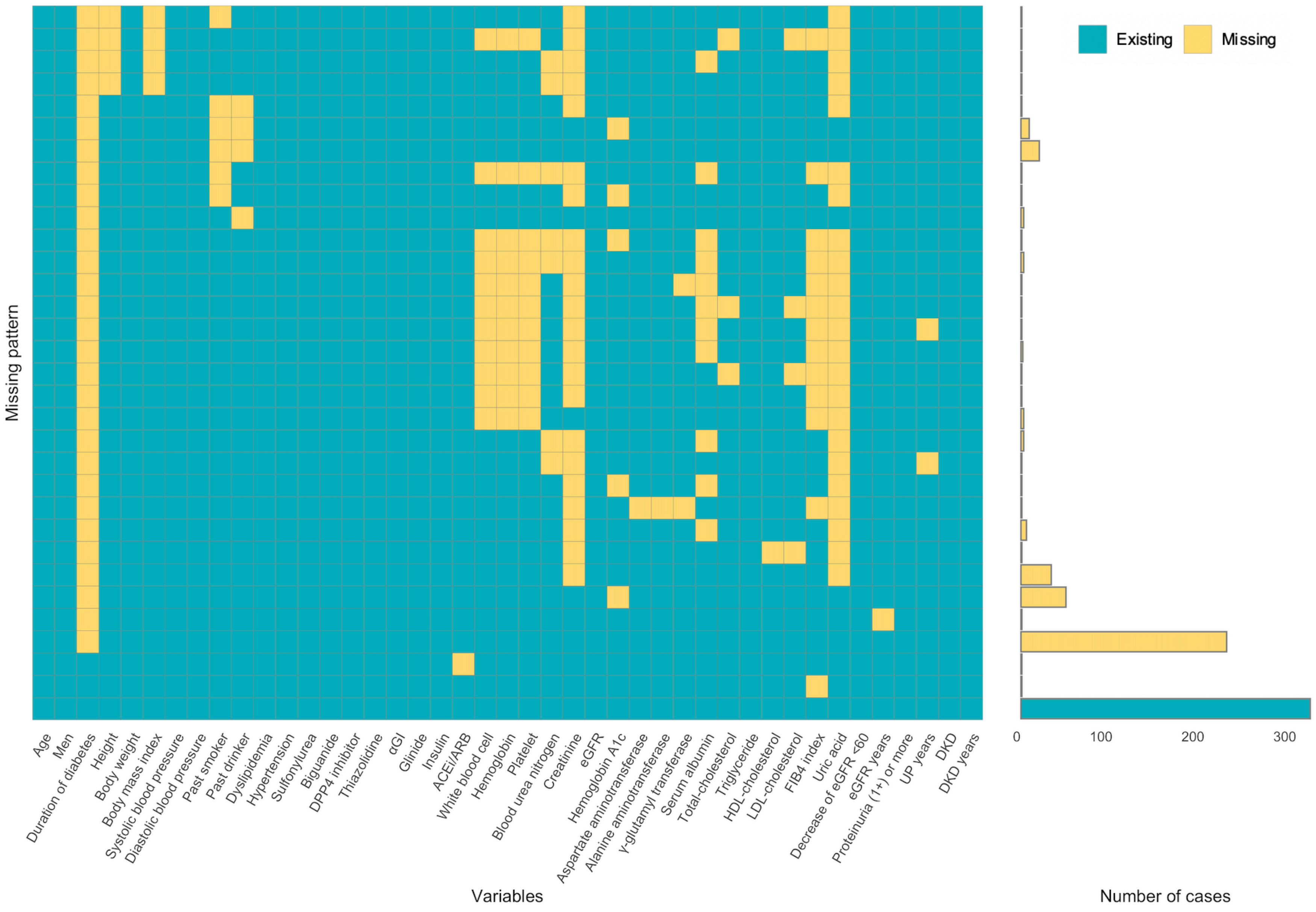

Supplement Figure 4. Pattern of missing data. In the left panel, green and yellow represents missing and existing values in the dataset. Each row represents the missing pattern. In the right panel, the horizontal bars display the frequencies of the corresponding combinations of existing and missing variables. VIM package 5.1.1 and ggplot 2 3.3 running on R 3.6.3. were used for visualization of the missing pattern.
