# Supplementary material for: High FIB4 index is an independent risk factor of diabetic kidney disease in type 2 diabetes
Source: Sci Rep. 2021 Jun 3;11:11753. doi: 10.1038/s41598-021-88285-6 (PMC8175689; doi:10.1038/s41598-021-88285-6)
Supplement: Supplementary file 5 — Supplementary Figure 5. [file 41598_2021_88285_MOESM5_ESM.pdf]

High FIB4 index is an independent risk factor of diabetic kidney disease in type 2 diabetes

Haruka Saito[1], Hayato Tanabe[1], Akihiro Kudo[1], Noritaka Machii[1], Moritake Higa[2], Satoshi Yamaguchi[1,5], Gulinu Maimaituxun[1], Kazumichi Abe[3], Atsushi Takahashi[3], Kenichi Tanaka[4], Koichi Asahi[6], Hiroaki Masuzaki[7], Hiromasa Ohira[3], Junichiro J. Kazama[4] and Michio Shimabukuro[1*]

[1]Department of Diabetes, Endocrinology and Metabolism, [3]Department of Gastroenterology and [4]Department of Nephrology and Hypertension, Fukushima Medical University, Fukushima, Japan; [2]Department of Diabetes and Lifestyle-Related Disease Center, Tomishiro Central Hospital, Okinawa, Japan; [5]Department of Cardiology, Nakagami Hospital, Okinawa, Japan; [6]Division of Nephrology and Hypertension, Iwate Medical University, Iwate, Japan; [7]Division of Endocrinology, Diabetes and Metabolism, Hematology, Rheumatology (Second Department of Internal Medicine), University of the Ryukyu, Okinawa, Japan.

*Corresponding author at: Department of Diabetes, Endocrinology and Metabolism, Fukushima Medical University, 1 Hikarigaoka, Fukushima City, Fukushima 960-1295, Japan; E-mail address: mshimabukuro-ur@umin.ac.jp (M. Shimabukuro); Tel +81-24-547-1305 , Fax +81-24-547-1311

A. DKD

	Multivariate	P value
FIB4 Index > 1.3 (yes vs. no)	1.513(1.139-2.011)	0.006
Age (per year)	1.003(0.989-1.018)	0.668
Sex (male vs. female)	1.132(0.825-1.552)	0.451
BMI (kg/m2)	1.009(0.989-1.029)	0.369
eGFR (mL/min/1.73 m2)	0.991(0.983-0.999)	0.036
HbA1c (%)	1.045(0.986-1.107)	0.146
Hypertension (yes vs. no)	1.323(1.014-1.726)	0.039
Dyslipidemia (yes vs. no)	1.009(0.773-1.319)	0.946
Past drinker (yes vs. no)	0.91(0.681-1.216)	0.537
Past smoker (yes vs. no)	1.159(0.888-1.51)	0.306
Sulfonylurea (yes vs. no)	1.437(1.037-1.991)	0.030
Biganide (yes vs. no)	1.027(0.72-1.464)	0.886
Thiazolidine (yes vs. no)	0.805(0.459-1.412)	0.449
αGI (yes vs. no)	0.78(0.524-1.163)	0.223
Insulin (yes vs. no)	1.559(1.002-2.425)	0.050
RAS Inhibitor (yes vs. no)	1.267(0.945-1.698)	0.115

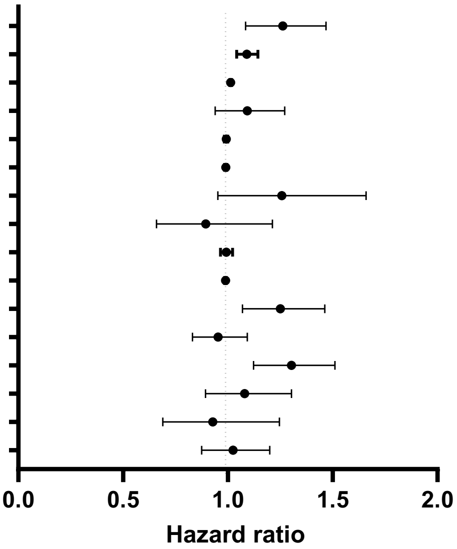

B. eGFR < 60

	Multivariate	P value
FIB4 Index > 1.3 (yes vs. no)	1.16(0.815-1.652)	0.424
Age (per year)	1.033(1.014-1.053)	0.001
Sex (male vs. female)	0.827(0.557-1.228)	0.353
BMI (kg/m2)	1.02(0.996-1.045)	0.110
eGFR (mL/min/1.73 m2)	0.956(0.943-0.969)	0.000
HbA1c (%)	1.094(1.017-1.177)	0.016
Hypertension (yes vs. no)	1.498(1.051-2.134)	0.025
Dyslipidemia (yes vs. no)	1.118(0.793-1.575)	0.525
Past drinker (yes vs. no)	0.912(0.629-1.322)	0.630
Past smoker (yes vs. no)	1.017(0.716-1.445)	0.826
Sulfonylurea (yes vs. no)	1.641(1.114-2.42)	0.012
Biganide (yes vs. no)	1.276(0.852-1.91)	0.238
Thiazolidine (yes vs. no)	0.865(0.423-1.772)	0.692
αGI (yes vs. no)	0.785(0.491-1.253)	0.310
Insulin (yes vs. no)	1.42(0.837-2.409)	0.195
RAS Inhibitor (yes vs. no)	1.291(0.913-1.826)	0.149

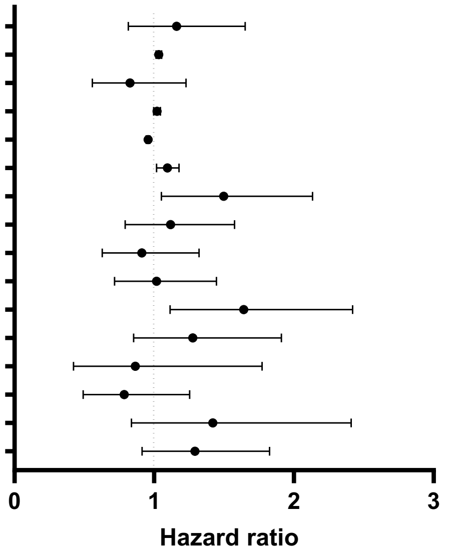

C. Proteinuria

	Multivariate	P value
FIB4 Index > 1.3 (yes vs. no)	1.489(1.049-2.112)	0.031
Age (per year)	0.992(0.974-1.01)	0.386
Sex (male vs. female)	1.279(0.85-1.924)	0.238
BMI (kg/m2)	1.011(0.986-1.036)	0.401
eGFR (mL/min/1.73 m2)	1.008(1.000-1.016)	0.050
HbA1c (%)	1.049(0.974-1.13)	0.218
Hypertension (yes vs. no)	1.213(0.868-1.697)	0.260
Dyslipidemia (yes vs. no)	0.993(0.709-1.391)	0.969
Past drinker (yes vs. no)	1.002(0.703-1.429)	0.859
Past smoker (yes vs. no)	1.406(1.000-1.977)	0.053
Sulfonylurea (yes vs. no)	1.401(0.94-2.087)	0.098
Biganide (yes vs. no)	1.05(0.674-1.636)	0.831
Thiazolidine (yes vs. no)	1.496(0.825-2.71)	0.185
αGI (yes vs. no)	0.844(0.517-1.377)	0.497
Insulin (yes vs. no)	1.34(0.763-2.353)	0.310
RAS Inhibitor (yes vs. no)	1.313(0.906-1.902)	0.152

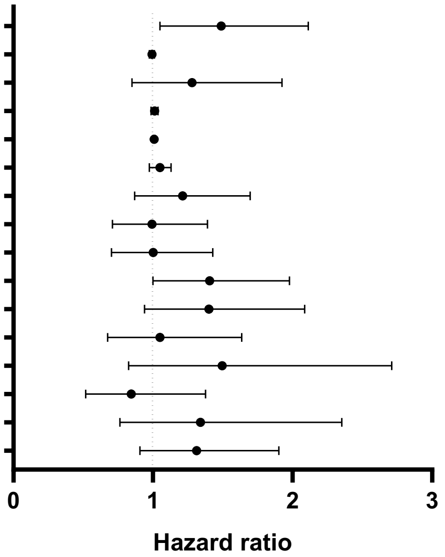

Supplement Figure 5. Univariate and Cox proportional hazard ratios of FIB4 index >1.30 for the development of (A) diabetic kidney disease (DKD: eGFR < 60 mL/min/1.73 m2 or proteinuria), (B) eGFR < 60 mL/min/1.73 m2, and (C) proteinuria in type 2 diabetic patients of a new virtual database by multiple imputation method for missing data. Cox proportional hazard models were adjusted for age, sex, BMI, baseline HbA1c, baseline eGFR, smoking and drinking status (current or past), comorbidities (hypertension, dyslipidemia) and anti-diabetic and anti-hypertensive medications. 95% CI, 95% confidence interval. n=691
