# Supplementary material for: High FIB4 index is an independent risk factor of diabetic kidney disease in type 2 diabetes
Source: Sci Rep. 2021 Jun 3;11:11753. doi: 10.1038/s41598-021-88285-6 (PMC8175689; doi:10.1038/s41598-021-88285-6)
Supplement: Supplementary file 6 — Supplementary Figure 6. [file 41598_2021_88285_MOESM6_ESM.pdf]

High FIB4 index is an independent risk factor of diabetic kidney disease in type 2 diabetes

Haruka Saito[1], Hayato Tanabe[1], Akihiro Kudo[1], Noritaka Machii[1], Moritake Higa[2], Satoshi Yamaguchi[1,5], Gulinu Maimaituxun[1], Kazumichi Abe[3], Atsushi Takahashi[3], Kenichi Tanaka[4], Koichi Asahi[6], Hiroaki Masuzaki[7], Hiromasa Ohira[3], Junichiro J. Kazama[4] and Michio Shimabukuro[1*]

[1]Department of Diabetes, Endocrinology and Metabolism, [3]Department of Gastroenterology and [4]Department of Nephrology and Hypertension, Fukushima Medical University, Fukushima, Japan; [2]Department of Diabetes and Lifestyle-Related Disease Center, Tomishiro Central Hospital, Okinawa, Japan; [5]Department of Cardiology, Nakagami Hospital, Okinawa, Japan; [6]Division of Nephrology and Hypertension, Iwate Medical University, Iwate, Japan; [7]Division of Endocrinology, Diabetes and Metabolism, Hematology, Rheumatology (Second Department of Internal Medicine), University of the Ryukyu, Okinawa, Japan.

*Corresponding author at: Department of Diabetes, Endocrinology and Metabolism, Fukushima Medical University, 1 Hikarigaoka, Fukushima City, Fukushima 960-1295, Japan; E-mail address: mshimabukuro-ur@umin.ac.jp (M. Shimabukuro); Tel +81-24-547-1305 , Fax +81-24-547-1311

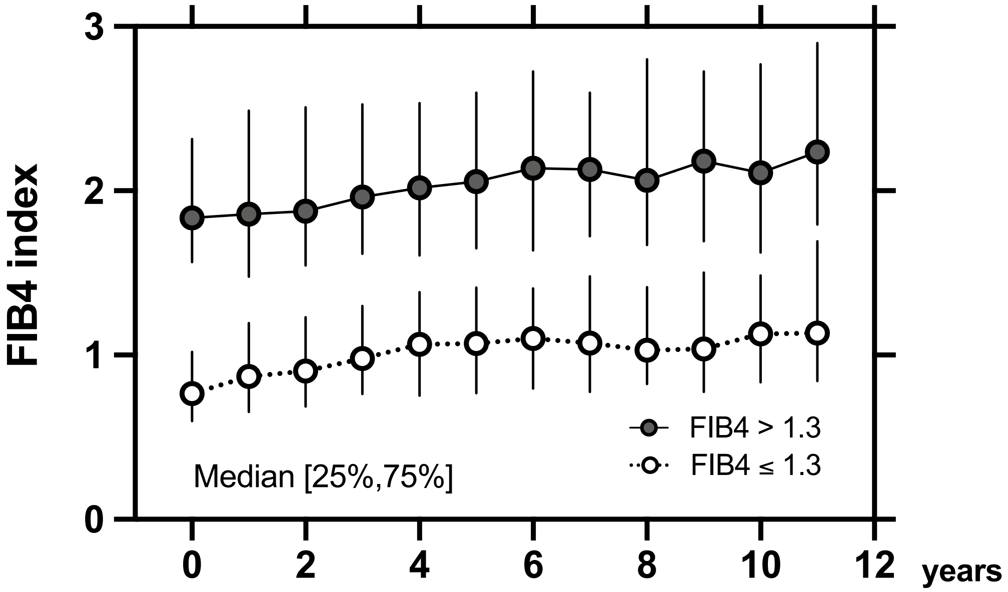

		Baseline	1	2	3	4	5	6	7	8	9	10	11
FIB4 >1.30	Median	1.84	1.86	1.88	1.96	2.02	2.06	2.14	2.13	2.06	2.18	2.11	2.24
	25%	1.57	1.48	1.55	1.62	1.61	1.65	1.64	1.72	1.67	1.69	1.62	1.79
	75%	2.32	2.49	2.51	2.53	2.53	2.60	2.73	2.60	2.80	2.73	2.77	2.90
FIB4 ≤1.30	Median	0.77	0.87	0.90	0.98	1.07	1.07	1.10	1.07	1.03	1.04	1.13	1.13
	25%	0.60	0.65	0.69	0.76	0.75	0.77	0.80	0.78	0.82	0.78	0.83	0.84
	75%	1.02	1.20	1.23	1.30	1.38	1.41	1.41	1.48	1.41	1.50	1.48	1.69

Supplement Figure 6. Annual changes in FIB4 index during the observational periods in FIB4 index >1.30 or ≤1.30. Values are median [25%, 75%].
