# Supplementary material for: High FIB4 index is an independent risk factor of diabetic kidney disease in type 2 diabetes
Source: Sci Rep. 2021 Jun 3;11:11753. doi: 10.1038/s41598-021-88285-6 (PMC8175689; doi:10.1038/s41598-021-88285-6)
Supplement: Supplementary file 7 — Supplementary Table. [file 41598_2021_88285_MOESM7_ESM.pdf]

High FIB4 index is an independent risk factor of diabetic kidney disease in type 2 diabetes

Haruka Saito, Hayato Tanabe, Akihiro Kudo, Noritaka Machii, Moritake Higa, Gulinu Maimaituxun, Kazumichi Abe, Atsushi Takahashi, Kenichi Tanaka, Koichi Asahi, Hiroaki Masuzaki, Hiromasa Ohira, Junichiro J. Kazama and Michio Shimabukuro

Supprement Table. The optimal cutoff point of FIB4 index by the highest Youden index for diabetic kideny disease (DKD)

	FIB4 index cutoff	Sensitivity	Specificity	AUC
DKD	1.296	0.398	0.740	0.566 (0.520-0.613)
eGFR < 60 mL/min/1.73 m^2	1.095	0.595	0.586	0.603 (0.554-0.651)
Proteinuria +	1.197	0.461	0.638	0.528 (0.475-0.581)

The optimal cutoff point of FIB4 index by the highest Youden index (EZR 1.40, Kanda Y. Investigation of the freely available easy-to-use software 'EZR' for medical statistics. Bone Marrow Transplant 2013; 48: 452-458, doi:10.1038/bmt.2012.244). Interestingly, the optimal cutoff points of FIB4 index especially for
