# Supplementary material for: High FIB4 index is an independent risk factor of diabetic kidney disease in type 2 diabetes
Source: Sci Rep. 2021 Jun 3;11:11753. doi: 10.1038/s41598-021-88285-6 (PMC8175689; doi:10.1038/s41598-021-88285-6)
Supplement: Supplementary file 8 — Supplementary Information. [file 41598_2021_88285_MOESM8_ESM.pdf]

High FIB4 index is an independent risk factor of diabetic kidney disease in type 2 diabetes

Haruka Saito[1], Hayato Tanabe[1], Akihiro Kudo[1], Noritaka Machii[1], Moritake Higa[2], Satoshi Yamaguchi[1,5], Gulinu Maimaituxun[1], Kazumichi Abe[3], Atsushi Takahashi[3], Kenichi Tanaka[4], Koichi Asahi[6], Hiroaki Masuzaki[7], Hiromasa Ohira[3], Junichiro J. Kazama[4] and Michio Shimabukuro[1*]

[1]Department of Diabetes, Endocrinology and Metabolism, [3]Department of Gastroenterology and [4]Department of Nephrology and Hypertension, Fukushima Medical University, Fukushima, Japan; [2]Department of Diabetes and Lifestyle-Related Disease Center, Tomishiro Central Hospital, Okinawa, Japan; [5]Department of Cardiology, Nakagami Hospital, Okinawa, Japan; [6]Division of Nephrology and Hypertension, Iwate Medical University, Iwate, Japan; [7]Division of Endocrinology, Diabetes and Metabolism, Hematology, Rheumatology (Second Department of Internal Medicine), University of the Ryukyu, Okinawa, Japan.

*Corresponding author at: Department of Diabetes, Endocrinology and Metabolism, Fukushima Medical University, 1 Hikarigaoka, Fukushima City, Fukushima 960-1295, Japan; E-mail address: mshimabukuro-ur@umin.ac.jp (M. Shimabukuro); Tel +81-24-547-1305 , Fax +81-24-547-1311

STROBE Statement—checklist of items that should be included in reports of observational studies

	Item No.	Recommendation	Page No.	Relevant text from manuscript
**Title and abstract**	1	(*a*) Indicate the study's design with a commonly used term in the title or the abstract	2	we evaluated the prognostic impact of FIB4 index on the risk of developing diabetic kidney disease (DKD) in Japanese patients with type 2 diabetes in a retrospective cohort study.
		(*b*) Provide in the abstract an informative and balanced summary of what was done and what was found	2	Nonalcoholic fatty liver disease (NAFLD) and nonalcoholic steatohepatitis (NASH) may be linked to development of chronic kidney diseases (CKD). The FIB4 index, a noninvasive liver fibrosis score, has been reported to predict CKD in non-diabetic patients, but there are no reports yet in diabetic cases. Therefore, we evaluated the prognostic impact of FIB4 index on the risk of developing diabetic kidney disease (DKD) in Japanese patients with type 2 diabetes in a retrospective cohort study. We assessed patients with type 2 diabetes with an eGFR ≥60 mL/min/1.73 m2 and without dipstick positive proteinuria (≥1+) at their first visit to our

department. Participants were divided into two groups based on the FIB4 index at their first visit: FIB4 index >1.3 and FIB4 index ≤1.3. The primary endpoint was defined as a decrease in eGFR <60 mL/min/1.73 m2 or the onset of proteinuria during the course of treatment. The average age of all 584 type 2 diabetic participants (360 [61.6%] men) was 55 ± 11 years. There were 187 patients in the FIB4 group >1.3 (32.0%) and the median observation period was 6.0 (3.8 – 11.0) years. Kaplan-Meier survival analysis indicated that the risks of developing DKD, eGFR <60 and proteinuria were all higher in FIB4 >1.3 patients than in FIB4 ≤1.3 patients. In the Cox regression analysis, an FIB4 index >1.3 was a significant predictor for onset of DKD (HR 1.54, 95% CI: 1.15-2.08) and proteinuria (HR 1.55, 95% CI: 1.08-2.23), but not for an eGFR <60 (HR 1.14, 95% CI: 0.79-1.99). To the best of our knowledge, this is the first study to demonstrate that an

				FIB4 index >1.3 has a prognostic impact on the development of CKD and proteinuria in type 2 diabetic patients. This warrants further investigation of the prognostic impact of the development of DKD or proteinuria.
**Introduction**				
Background/rationale	2	Explain the scientific background and rationale for the investigation being reported	3	Chronic kidney disease (CKD) associated with diabetes mellitus, often referred as diabetic kidney disease (DKD), is the leading cause of end-stage kidney disease (ESKD) for patients with diabetes. The treatment of earlier stages of DKD is effective in slowing the progression toward ESRD. Thus, early detection of precursors and/or risk factors for DKD is crucial. Family history of DKD, smoking history, and control of glycemic, blood pressure, and plasma lipid levels are established factors for identifying people at a greater risk of DKD development and progression. Among emerging risk markers for CKD, nonalcoholic fatty liver disease

				(NAFLD) is also an exacerbation factor for the development and progression of CKD in the non-diabetic and diabetic populations. For this reason, multiple scoring systems that noninvasively predict the progression to NASH and liver fibrosis have been proposed. The FIB4 index is a high ability non-invasive scoring system used to predict NASH and liver fibrosis. A relationship between the FIB4 index and onset of CKD was reported in non-diabetic patients, but the relationship has never been studied in a diabetic population.
Objectives	3	State specific objectives, including any prespecified hypotheses	3	Therefore, we evaluated the prognostic impact of the FIB4 index on the risk of developing DKD in Japanese type 2 diabetic patients in a multi-center retrospective cohort study.
**Methods**				
Study design	4	Present key elements of study design early in the paper	7	This is an observational retrospective cohort study.
Setting	5	Describe the setting, locations, and relevant dates, including periods of recruitment, exposure, follow-up, and data collection	7-8	Inclusion criteria of the participants was: adult patients

				with type 2 diabetes mellitus who had visited the Department of Diabetes, Endocrinology, and Metabolism, Fukushima Medical University Hospital or Department of Diabetes and Lifestyle-Related Disease Center, Tomishiro Central Hospital between January 2002 and March 2019. Written informed consent was obtained from the patients between January 2018 and March 2019 in the Department of Diabetes, Endocrinology, and Metabolism, School of Medicine, Fukushima Medical University Hospital and informed consent for participants in Tomishiro Central Hospital was waived by the Tomishiro Central Hospital Ethics Committee.
Participants	6	(*a*) *Cohort study*—Give the eligibility criteria, and the sources and methods of selection of participants. Describe methods of follow-up<br>*Case-control study*—Give the eligibility criteria, and the sources and methods of case ascertainment and control selection. Give the rationale for the choice of cases and controls<br>*Cross-sectional study*—Give the eligibility criteria, and the sources and methods of selection of participants	8	Total of 1,197 patients with type 2 diabetes mellitus were selected from both hospitals on their medical records. On the below definition of DKD, 279 CKD/DKD at baseline were excluded. On exclusion criteria, 81 were removed by complications of liver, kidney

and hematologic diseases (Figure 1). Twenty-four patients with non-diabetic kidney diseases (chronic glomerulonephritis, vasculitis, polycystic kidney disease, and renal cancer) and 47 patients with liver disease other than NAFLD (viral hepatitis, autoimmune liver disease, liver transplantation). Patients diagnosed with liver cirrhosis and heavy drinker (consumption of ethanol less than 20 g/day for women and 30 g/day for men) had been excluded in advance. After deleted for 146 with observation period <1 year and 107 with missing data, the remaining 584 patients with type 2 diabetes mellitus were enrolled and their paper and/or electrical medical records were scrutinized from October 2002 to March 2019. Their first visit to either hospitals was considered as the baseline. The parameters such as age, sex, history of diabetes, family and social history, medical checkup history, complications, medications, laboratory data,

				and all dates were recorded.
		(*b*) *Cohort study*—For matched studies, give matching criteria and number of exposed and unexposed<br>*Case-control study*—For matched studies, give matching criteria and the number of controls per case		
Variables	7	Clearly define all outcomes, exposures, predictors, potential confounders, and effect modifiers. Give diagnostic criteria, if applicable	8	The primary endpoint of this study was onset of DKD. The secondary endpoint of this study was each onset of eGFR <60 mL/min/1.73 m2 or proteinuria 1+ with a dipstick urine test.
Data sources/ measurement	8*	For each variable of interest, give sources of data and details of methods of assessment (measurement). Describe comparability of assessment methods if there is more than one group	8-9	The FIB4 index was calculated by Age (year) × AST (IU/L) / (√ ALT (IU/L) × Platelet count (109/L)) 29,47. A cut-off value of 1.3 or less, which was 90% negative for the progression of liver fibrosis, was applied. Hypertension was defined as systolic blood pressure ≥140 mmHg or diastolic blood pressure ≥90 mmHg or those taking antihypertensive drugs. Dyslipidemia was defined as LDL cholesterol ≥140 mg/dL or those taking antihyperlipidemic drugs.
Bias	8	Describe any efforts to address potential sources of bias		Figure 5. There are limitations in this study. First, since a liver biopsy was not performed, the

				correlation between the FIB4 index and the actual degree of fibrosis is not objective. Second, this was a retrospective cohort study and the causal or correlation relationship cannot be determined in this study. Third, this study comprised of only Japanese race from only two centers, suggesting a possibility of selection bias. Fourth, it could be arguable that respective assessment of "proteinuria" and "worsening eGFR" are clinically relevant or not 4. Since progression of proteinuria is the main driver of the DKD, it might be meaningless to differentiate "proteinuria" and "worsening eGFR" separately.
Study size	10	Explain how the study size was arrived at	7-8	adult patients with type 2 diabetes mellitus who had visited the Department of Diabetes, Endocrinology, and Metabolism, Fukushima Medical University Hospital or Department of Diabetes and Lifestyle-Related Disease Center, Tomishiro Central Hospital between January 2002 and March 2019.

Continued on next page

Quantitative variables	11	Explain how quantitative variables were handled in the analyses. If applicable, describe which groupings were chosen and why	8-9	The FIB4 index was calculated by Age (year) × AST (IU/L) / ($\sqrt{}$ ALT (IU/L) × Platelet count (109/L)) 29,47. A cut-off value of 1.3 or less, which was 90% negative for the progression of liver fibrosis, was applied.
Statistical methods	12	(*a*) Describe all statistical methods, including those used to control for confounding	10-11	Continuous and parametric values are expressed as mean ± standard deviation, and the nonparametric variables as median (first quartile-third quartile). The two-tailed unpaired student's t-test and Mann-Whitney *U* test were used for parametric and non-parametric data, respectively. Categorical variables are shown as percentages and were analyzed using the Chi-square test. Univariate survival analysis was calculated using the Kaplan-Meier curve and analyzed by a log rank test. Univariate and Cox proportional hazards model were used to determine the independent contributions of the FIB4 index as a dichotomizing variable (> 1.3 vs. ≤ 1.3) to the development of DKD, a decline in eGFR (<60 mL/min/1.73 m^2), or proteinuria after adjusting for age, sex, BMI, baseline HbA1c, baseline eGFR, smoking and drinking status (current or past), comorbidities (hypertension, dyslipidemia) and anti-diabetic and anti-hypertensive medications. Covariates used for the Cox proportional hazards model were chosen from possible confounding factors for DKD[1-4].<br>For sensitivity analyses: univariate and Cox proportional hazards models were repeated: 1) by using the FIB4 index as continuous or quartile variable≈; 2) by HbA1c as a time dependent cobariate plus possible emerging biomarker for DKD (white blood cell count); 3) by a new data-set with multiple imputation method for missing data analysis: 4) time dependent AUC of FIB4 Index for the develeopme of DKD, eGFR < 60 and proteinuria.<br>Values of *P* <0.05 were considered as statistically significant. Statistical analyses were conducted using SPSS version 25 (SPSS, Inc., Chicago, Illinois, USA) or R 3.6.3. VIM package 5.1.1 and ggplot2

				3.3 running on R 3.6.3 are used for visualization of the missing pattern.
		(*b*) Describe any methods used to examine subgroups and interactions	10-11	For sensitivity analyses: univariate and Cox proportional hazards models were repeated: 1) by using the FIB4 index as continuous or quartile variable≈; 2) by HbA1c as a time dependent cobariate plus possible emerging biomarker for DKD (white blood cell count); 3) by a new data-set with multiple imputation method for missing data analysis: 4) time dependent AUC of FIB4 Index for the develeopme of DKD, eGFR < 60 and proteinuria.
		(*c*) Explain how missing data were addressed	1-11	For sensitivity analyses: univariate and Cox proportional hazards models were repeated: 1) by using the FIB4 index as continuous or quartile variable≈; 2) by HbA1c as a time dependent cobariate plus possible emerging biomarker for DKD (white blood cell count); 3) by a new data-set with multiple imputation method for missing data analysis: 4) time dependent AUC of FIB4 Index for the develeopme of DKD, eGFR < 60 and proteinuria.
		(*d*) *Cohort study*—If applicable, explain how loss to follow-up was addressed<br>*Case-control study*—If applicable, explain how matching of cases and controls was addressed<br>*Cross-sectional study*—If applicable, describe analytical methods taking account of sampling strategy		
		(*e*) Describe any sensitivity analyses	10-11	For sensitivity analyses: univariate and Cox proportional hazards models were repeated: 1) by using the FIB4 index as continuous or quartile variable≈; 2) by HbA1c as a time dependent cobariate plus possible emerging biomarker for DKD (white blood cell count); 3) by a new data-set with multiple imputation method for missing data analysis: 4) time dependent AUC of FIB4 Index for the develeopme of DKD, eGFR < 60 and proteinuria.
**Results**				
Participants	13*	(a) Report numbers of individuals at each stage of study—eg numbers potentially eligible, examined for eligibility, confirmed	3	

		eligible, included in the study, completing follow-up, and analysed	
		(b) Give reasons for non-participation at each stage	
		(c) Consider use of a flow diagram	Figure1
Descriptive data	14*	(a) Give characteristics of study participants (eg demographic, clinical, social) and information on exposures and potential confounders	Table1
		(b) Indicate number of participants with missing data for each variable of interest	Figure1
		(c) *Cohort study*—Summarise follow-up time (eg, average and total amount)	Table1
Outcome data	15*	*Cohort study*—Report numbers of outcome events or summary measures over time	Table1
		Case-control study—Report numbers in each exposure category, or summary measures of exposure	
		Cross-sectional study—Report numbers of outcome events or summary measures	
Main results	16	(*a*) Give unadjusted estimates and, if applicable, confounder-adjusted estimates and their precision (eg, 95% confidence interval). Make clear which confounders were adjusted for and why they were included	Figure3
		(*b*) Report category boundaries when continuous variables were categorized	
		(*c*) If relevant, consider translating estimates of relative risk into absolute risk for a meaningful time period	

Continued on next page

Other analyses	17	Report other analyses done—eg analyses of subgroups and interactions, and sensitivity analyses		
**Discussion**				
Key results	18	Summarise key results with reference to study objectives	4	
Limitations	19	Discuss limitations of the study, taking into account sources of potential bias or imprecision. Discuss both direction and magnitude of any potential bias	7	
Interpretation	20	Give a cautious overall interpretation of results considering objectives, limitations, multiplicity of analyses, results from similar studies, and other relevant evidence		
Generalisability	21	Discuss the generalisability (external validity) of the study results		
**Other information**				
Funding	22	Give the source of funding and the role of the funders for the present study and, if applicable, for the original study on which the present article is based	10	Funding This study was supported by Japan Society for the Promotion of Science (JPSP) (Grant Number JP16K01823 to M.S. and JP17K00924 to A.K and M.S.) and a grant from Japan Agency for Medical Research and Development (AMED, 965304 to M.S.)

*Give information separately for cases and controls in case-control studies and, if applicable, for exposed and unexposed groups in cohort and cross-sectional studies.

Note: An Explanation and Elaboration article discusses each checklist item and gives methodological background and published examples of transparent reporting. The STROBE checklist is best used in conjunction with this article (freely available on the Web sites of PLoS Medicine at http://www.plosmedicine.org/, Annals of Internal Medicine at http://www.annals.org/, and Epidemiology at http://www.epidem.com/). Information on the STROBE Initiative is available at www.strobe-statement.org.
